# ﻿New species of the Spiny Mouse genus *Neacomys* (Cricetidae, Sigmodontinae) from northwestern Ecuador

**DOI:** 10.3897/zookeys.1175.106113

**Published:** 2023-08-17

**Authors:** Nicolás Tinoco, Claudia Koch, Javier E. Colmenares-Pinzón, Francisco X. Castellanos, Jorge Brito

**Affiliations:** 1 Sección de Mastozoología, Museo de Zoología, Facultad de Ciencias Exactas y Naturales, Pontificia Universidad Católica del Ecuador, Quito, Ecuador Instituto Nacional de Biodiversidad (INABIO) Quito Ecuador; 2 Fundación Great Leaf, Quito, Pichincha, Ecuador Pontificia Universidad Católica del Ecuador Quito Ecuador; 3 Instituto Nacional de Biodiversidad (INABIO), Pasaje Rumipamba 341 y Av. de los Shyris, PB 17-07-8976, Quito, Ecuador Fundación Great Leaf Quito Ecuador; 4 Leibniz Institute for the Analysis of Biodiversity Change/Museum Koenig, Bonn, Germany Leibniz Institute for the Analysis of Biodiversity Change/Museum Koenig Bonn Germany; 5 Grupo de Estudios en Biodiversidad, Escuela de Biología, Universidad Industrial de Santander, Carrera 27 # 9, Bucaramanga, Colombia Universidad Industrial de Santander Bucaramanga Colombia; 6 Department of Biological Sciences, Texas Tech University, Lubbock, Texas, USA Texas Tech University Lubbock United States of America

**Keywords:** Chocó biogeographic, *Neacomystenuipes*, premontane forest

## Abstract

*Neacomys* is a genus of small spiny or bristly sigmodontine rodents that are common components of mammalian faunas in multiple biomes on Central and South America. Recent studies on this group have demonstrated that there is cryptic diversity yet to be discovered within currently recognized species that have not received comprehensive revisions, as well as in areas that have been overlooked. Here we ratify this assertion by describing a new species previously misidentified as the Narrow-footed Spiny Mouse (*Neacomystenuipes*) from the Chocó biogeographic region in northwestern Ecuador, *Neacomysmarci* Brito & Tinoco, **sp. nov.** Distinctiveness of this entity is supported by the combination of the following morphological characters: small size (head-body length 65–85 mm); long tail (69–126% longer than head-body length); pale buff-colored but gray-based belly fur; white throat; hypothenar pad usually absent; long nasals; and a condylar process higher than the coronoid process. Likewise genetic distance analyses and phylogenetic reconstructions based on cytochrome-b (Cytb) sequence data indicate a clear divergence from typical populations of *N.tenuipes*, and a sister relationship between them. The results presented here increase the diversity of *Neacomys* to 24 species, placing it among the most diverse genera within the sigmodontine rodents.

## ﻿Introduction

*Neacomys* is a widely distributed genus of small spiny or bristly rodents that collectively occupy representative regions and habitats in easternmost Panama and the northern half of South America (Patton et al. 2015; Pardiñas et al. 2017; Caccavo and Weksler 2021; Semedo et al. 2021). Currently, 23 species are recognized within this group, occurring its highest concentration in the rainforests of the Amazon region (Hurtado and Pacheco 2017; Semedo et al. 2020, 2021; Brito et al. 2021a; Caccavo and Weksler 2021).

From the years 2017 through 2021, taxonomy of *Neacomys* has been remarkably dynamic and has resulted in the description of 11 species (Hurtado and Pacheco 2017; Sanchez-Vendizú et al. 2018; Semedo et al. 2020, 2021; Brito et al. 2021a; Caccavo and Weksler 2021; Colmenares-Pinzon 2021). The progress in the understanding of its diversity has been mainly achieved thanks to the exhaustive revision of material deposited in museum collections (Semedo et al. 2020, 2021; Caccavo and Weksler 2021), as well as increased collection efforts and implementation of molecular analyses (Brito et al. 2021a; Colmenares-Pinzon 2021). However, as there are still many unexplored areas in the heterogenous geography of South America and adjacent Central America (Panama), some of the currently recognized species have not undergone comprehensive taxonomic evaluations, and it is possible that the real diversity of the genus is underestimated.

The Chocó Biogeographic region is considered one of the most diverse hotspots in South America (Myers et al. 2000), yet one of the least studied regions for *Neacomys* despite its large extension (along the Pacific coasts of Panama, Colombia, and Ecuador). To date, only two species are known to occur in the Chocó, the Painted Bristly Mouse *N.pictus*, and the Narrow-footed Spiny Mouse *N.tenuipes* (Patton et al. 2015; Pardiñas et al. 2017). The former has been recorded from one locality in Panama (Goldman 1912), and is scarcely represented in museums, by fewer than 12 specimens collected more than 30 years ago (VertNet Database): the presence of *N.tenuipes* is supported by only three specimens from two localities in Colombia (Colmenares-Pinzon 2021), and by an unclear number of specimens from at least four localities in Ecuador (Jarrín 2001; Brito et al. 2021a). Poor knowledge about the distribution of *Neacomys* throughout the Chocó region is accompanied by a lack of genetic characterization. This has prevented the inclusion of *N.pictus* in phylogenetic analyses of the genus, and thus there are no clues about its relationship with respect to other species. In the case of the populations from the department of Cauca, Colombia, and those from Ecuador, this has precluded the possibility of addressing their degree of differentiation from typical *N.tenuipes*, or even determine if they represent different species as some authors have hypothesized [e.g., the first one has been treated as *N.pusillus* Allen, 1912 (Caccavo and Weksler 2021) whereas the second was treated as *N.pictus* Goldman, 1912 (Jarrín 2001)]. The uncertainty about the affinity of some populations to *N.tenuipes* illustrates a possible cause of an underestimated diversity within the genus, where some of the currently recognized species have not been reviewed in detail.

With the recent collections of several specimens resembling *Neacomystenuipes* in previously unexplored areas of northwestern Ecuador, their genetic and morphological characterization, and their comparison with material from different museums, this work describing a new species constitutes a forward step towards a better understanding of the variation within what has been considered a widely distributed and homogeneous species, as well as of the real diversity of the genus in the Chocó biogeographic region.

## ﻿Materials and methods

### ﻿Specimens

Specimens of *Neacomys* from northwestern Ecuador reviewed here were mostly obtained from field expeditions conducted by JB and his team to two protected areas. Reserva Dracula was sampled during three consecutive nights in November 2016, January 2017, and July 2017, respectively, using 10–12 pitfall traps (20–60 liters), which yielded an effort of 430 traps/night. On the other hand, Reserva Canandé was also surveyed with 20 pitfall traps, and with 100 standard Sherman traps (7.5 × 9 × 27 cm) during four consecutive nights in November 2020, and during six nights in October 2022. Capture effort with the Sherman traps was 1,030 traps/night. In all three cases, all traps were placed near runways, holes, and other signs of small mammal activity, and baited with rolled oats mixed with vanilla and alternating with concentrates cattle feed (Voss et al. 2001; Brito et al. 2020). All activities related to the handling and collection of specimens were conducted according to the protocols approved by the American Society of Mammalogists (Sikes et al. 2016). Research permits were issued by the Ecuadorian Ministry of Environment (MAE-DNB-CM-2019-0126, MAAE-ARSFC-2020-0642, and MAATE-ARSFC-2022-2583).

Mounted dry skins, skeletons, fluid-preserved bodies, and tissue samples stored in 96% ethanol were deposited in the biological collections of the Instituto Nacional de Biodiversidad (INABIO; Quito, Ecuador). Initially, specimens from both reserves were identified as *Neacomystenuipes* based on discrete morphological characters. Further comparisons were carried out between these specimens and additional material of the genus deposited in local and international mammal collections: Museo de la Escuela Politécnica Nacional (**MEPN**, Quito, Ecuador); Museo de Zoología de la Pontificia Universidad Católica del Ecuador (**QCAZ**, Quito, Ecuador); Instituto de Investigación de Recursos Biológicos Alexander von Humboldt (**IAvH-M**, Bogotá, Colombia); Museo de Historia Natural de la Universidad Industrial de Santander (**UIS-MHN-M**, Santander, Colombia); Museo de Historia Natural de la Universidad de Caldas (**MHN-UCa-M**, Caldas, Colombia) and Instituto Nacional de Biodiversidad (**MECN**, Quito, Ecuador). All studied material is listed in Appendix 1.

### ﻿Morphological qualitative and quantitative comparisons

For the craniodental morphological comparisons the terminology follows Patton et al. (2000), Voss et al. (2001), Hurtado and Pacheco (2017), Sánchez-Vendizú et al. (2018), Semedo et al. (2020), and Caccavo and Weksler (2021). The soft anatomy was reviewed considering the concepts in Carleton (1973) and Pardiñas et al. (2020).

A detailed structural scrutiny of the skull of one specimen (MECN 6232; Estación Fisher, Ecuador) was done using a high-resolution micro-computed tomography (micro-CT) desktop scanner device (Bruker SkyScan 1173, Kontich, Belgium) at the Leibniz Institute for the Analysis of Biodiversity Change/Museum Koenig (**LIB**, Bonn, Germany). To avoid movements during the scanning process, the material was placed in a small plastic container embedded in cotton wool. Acquisition parameters comprised: an X-ray beam (source voltage 43 kV and current 116 μA) without the use of a filter; 960 projections of 500 ms exposure time each with a frame averaging of 4 recorded over 180° using rotation steps of 0.25 degrees, resulting in a scan duration of 55 min 28 s; a magnification setup generating data with an isotropic voxel size of 12.07 μm. The CT-dataset was reconstructed with N-Recon software (Bruker MicroCT, Kontich, Belgium) and rendered in three dimensions using CTVox for Windows 64 bits v. 2.6 (Bruker MicroCT, Kontich, Belgium).

All specimens were classified into five age classes defined by Semedo et al. (2020) and Caccavo and Weksler (2021) based on the level of eruption of the third molar and the wear of the occlusal surface of the molars. Only specimens between ages 3 and 6 were used in the quantitative morphological comparisons.

For these comparisons, a total of four external and 19 craniodental measurements were considered according to: Carleton and Musser (1989); Patton et al. (2000); Voss et al. (2001); Hurtado and Pacheco (2017); Sánchez-Vendizú et al. (2018); Semedo et al. (2020); Brito et al. (2021a). Four body measurements: head and body length (**HBL**); tail length (**TL**); hind foot length (**HF**); ear height (**E**); and body mass (**w**, in grams, g); condyloincisive length (**CIL**); length of incisive foramina (**LIF**); breadth of incisive foramina (**BIF**); length of upper diastema (**LD**); crown length of maxillary toothrow (**LM**); alveolar width (**AW**); breadth of palatal bridge (**BPB**); length of rostrum (**LR**); length nasal (**LN**); rostral width (**RW-2**); least interorbital breadth (**LIB**); orbital length (**OL**); breadth of zygomatic plate (**BZP**); zygomatic breadth (**ZB**); braincase breadth (**BB**); occipital condyle breadth (**OCB**); basioccipital length (**BOL**); cranial depth (**CD**); breadth of the first upper molar (**BM1**).

We recorded external measurements from tags, and for the craniodental measurements we used digital calipers to the nearest 0.01 mm in all presumed specimens of *N.tenuipes* recently collected in northwestern Ecuador. We also measured older specimens of the species and other members of the genus housed in museums in Colombia and Ecuador (see above).

The craniodental measurements from 108 specimens tentatively identified as *N.tenuipes*, N.cf.pictus, and *N.rosalindae* Sánchez-Vendizú, Pacheco & Vivas-Ruiz, 2018 were compiled in a matrix with 2,376 values. This dataset was analyzed in R v. 4.2.1 (R Core Team 2022) and inferred for missing values using the missMDA package (Josse and Husson 2016). The iterative PCA algorithm was preferred for this purpose with a maximum of 1,000 iterations and a 1e-6 threshold to assess convergence. The estimated number of components needed to predict the missing values were obtained by running 100 simulations with the leave-one-out cross-validation method. Morphological characters were checked for high degrees of correlation using Spearman’s coefficient, yet none were discarded since correlation values were ≤ 0.95. Non-parametric methods were preferred in all analyses (Šlenker et al. 2022).

Multivariate analyses performed in this study included Principal Component (PCA), and the K nearest neighbor classificatory Discriminant Analyses (KNN) with the MorphoTools2 package (Šlenker et al. 2022). For the latter, samples were grouped a priori as follows: 1) recently collected samples from northwestern Ecuador presumably belonging to *N.tenuipes*; 2) older museum specimens from northeastern Ecuador presumably belonging to N.cf.pictus; 3) typical *N.tenuipes* from Colombia; 4) *N.rosalindae*. To ensure that only invariant and non-linear characters were used in the KNN analysis, a stepwise discriminant analysis was conducted first and selected the following subset of characters: OL, LR, AW, BPB, CIL, HBL, LM, E, TL, LD, and ZB. Neacomyscf.pictus specimens were excluded because the total number of individuals (*n* = 2) was smaller than the total number of analyzed characters (Šlenker et al. 2022). The KNN results were plotted by centering and scaling the two variables that contributed the most to the discrimination of groups as predicted by the R^2^ and F-values of the stepwise analysis.

Individuals’ classification prediction was done using nine neighbors (k = 9) by estimating Euclidean distances through a cross-validation method. The precision of the classification was finally obtained as a percentage by comparing the model’s prediction to the *a priori* classification herein assigned.

Statistical tests for non-uniformly distributed data were calculated and plotted using the ggstatsplot package (Patil 2021), to verify for significant statistical differences in the variables inferred to exert a greater effect on taxon differences. A Kruskal-Wallis test was applied to determine if the groups’ medians were significantly different, followed by a Dunn test for a pairwise comparison of groups adjusting the p-value with the Holm method to control for the family-wise error rate (Holm 1979).

### ﻿DNA extraction, amplification, and sequencing

DNA was extracted from muscle samples of the presumed specimens of *N.tenuipes* recently collected in northwestern Ecuador. The guanidine thiocinate protocol was used for DNA extraction (Bilton and Jaarola 1996). We amplified between 1000 and 1100 bases pair of mitochondrial gene Cytochrome b (Cytb); we used the forward primer MVZ05, and the reverse primers MVZ16H, MVZ14 (Smith and Patton 1993). The thermal profile for the amplification of Cytb included: an initial denaturation at 94 °C for 180 s, 35 cycles of denaturation at 94 °C for 45 s, primer annealing at 45 °C for 2 min, and the final elongation at 72 °C for 60s (Smith and Patton 1993; Bonvicino and Moreira 2001). The amplicons were sequenced at Macrogen Inc. in South Korea. The Cytb sequences were edited and assembled in the Geneious R11 program (https://www.geneious.com) and then verified to represent endogenous DNA of *Neacomys* by performing independent searches with the Basic Local Alignments Search Tool (BLAST) (Altschul et al. 1990).

### ﻿Phylogenetic analyses

We tried to include representatives of the 23 known *Neacomys* species (Appendix 2), including some sequences from other genera of sigmodontine rodents that were used as outgroups (Appendix 2). The algorithm CLUSTAL-W was used for this purpose as implemented in Geneious R11. The ML tree was inferred using IQ-TREE (Nguyen et al. 2015). The BI analysis was conducted with MrBayes 3.2 (Ronquist et al. 2012), on the CIPRES Science Gateway platform (Miller et al. 2010), the analysis was carried out with two runs and four chains, were run for 10,000,000 generations, with a sampling every 1,000 generations and a burn-in of 0.25. Convergence was evaluated by the effective sample size (EES) and the potential scale reduction factor (PSRF). For most of the parameters the EES should be ≥ 200 and for the PSRF most of the values of the parameters should be between 1.0 and 1.2.

### ﻿Genetic distances

We calculated an analysis of genetic divergence using an alignment restricted to the genus *Neacomys* obtained as described above. Uncorrected p-distances (intra and interspecific) were calculated with the MEGA X program (Kumar et al. 2018) and transformed to percentage values. The uncorrected p-distances were calculated in other works (Brito et al. 2021a; Colmenares-Pinzon; Semedo et al. 2020, 2021).

## ﻿Results

### ﻿Morphological qualitative and quantitative comparisons

Morphological qualitative revision and comparisons revealed that recently collected specimens and some older museum specimens from northwestern Ecuador are different from typical *Neacomystenuipes* from Colombia in multiple discrete characters.

**Figure 1.**
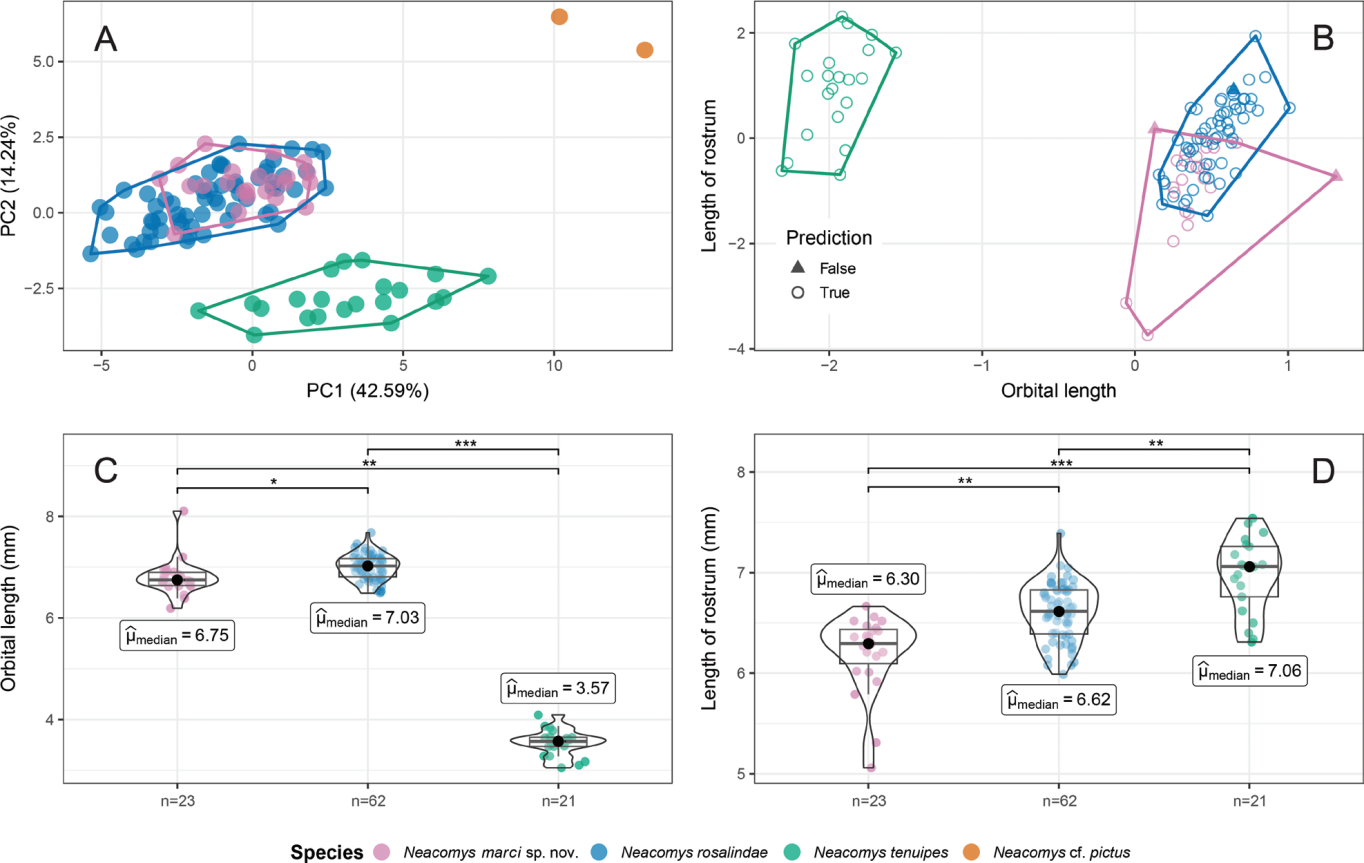
Morphometric and statistical analyses **A** scatterplots of the Principal Components **B** the K neighbor discriminant analyses. Each taxon is enclosed by a convex hull, and color codes are detailed in the legend **C, D** the distribution of the data is shown in a violin boxplot; the median of each taxon character is indicated with a black dot. Only statistically significant differences among taxa are shown with the *p-adjusted* Holm method (* p < 1e-3, ** p < 1e-4, *** p < 1e-9).

The two principal components of the PCA analysis explained 56.83% of the variation in the craniodental measurements, with CIL and RW-2 contributing to a greater extent to each one of them, respectively (Table 1). There was a clear overlap in the morphospace between recently collected samples from northwestern Ecuador and samples of the Rosalind’s bristly mouse, *N.rosalindae*. Older museum samples of N.cf.pictus, also from northwestern Ecuador, and typical samples of *N.tenuipes* from Colombia were recovered as two discrete groups (Fig. 1A). Likewise, typical *N.tenuipes* was completely discriminated in the KNN while recently collected samples (northwestern Ecuador) and *N.rosalindae* attained some degree of separation (Fig. 1B); the algorithm achieved accurate classification for samples from northwestern Ecuador, and *N.rosalindae*, with success rates of 91.3% and 95.8% respectively (Fig. 1B, Table 2).

**Table 1.** Results of the Principal Component Analysis (PCA). The overall contribution of each component is shown between parentheses, and the loadings with the highest absolute values in each component are bolded and displayed in rows. Character abbreviations are detailed in the text.

	PC1 (42.59%)	PC2 (14.24%)
HBL	0.1884563	0.113983562
TL	0.2082709	0.332087602
HF	0.2241264	0.201834179
E	0.1131424	0.207207965
CIL	**0.3033246**	-0.100529150
LIF	0.1912464	0.135697494
BIF	0.2025086	0.144585511
LD	0.2605398	-0.153473315
LM	0.2145628	0.119512958
AW	**0.2830731**	-0.049754832
BPB	0.1485273	-**0.217453585**
LR	0.1740795	0.138869902
LN	0.2325637	-0.007425074
RW-2	0.0203125	-**0.517612008**
LIB	0.2002134	-0.073239339
OL	0.1168325	-**0.486171390**
BZP	0.2230202	-0.171806332
ZB	**0.2882257**	-0.098729676
BB	0.2409027	-0.063978461
OCB	0.2474346	-0.078435817
BOL	0.2283143	-0.160505793
CD	0.1721603	-0.227526278

**Table 2.** Confusion matrix displaying the performance of the K nearest-neighbor classification for three *Neacomys* species. *n* represents the number of individuals used as input in the model. Values in “as species” columns represent the number of individuals assigned to each taxon, and the accuracy of the prediction is given as a percentage in the last column.

Taxon	*n*	as *N.marci* sp. nov.	as *N.rosalindae*	as *N.tenuipes*	correct (%)
*marci* sp. nov.	23	21	2	0	91.30
*rosalindae*	62	1	61	0	95.08
*tenuipes*	21	0	0	21	100.00
Total	106	22	63	21	97.17

The characters chosen by the stepwise analysis as the greatest contributors to morphologic discrimination were OL (R^2^ = 0.96; F = 1287.68; p < 1e-15) and LR (R^2^ = 0.52; F = 55.21; p < 1e-15). The Kruskal-Wallis test revealed that the medians significantly differed across all groups (p < 1e-5), and the Dunn pairwise test proved that both characters were significantly different between all species with p-adjusted < 0.001 (Fig. 1C, D). These results constitute additional evidence supporting differentiation of the recently collected Ecuadorian specimens from typical specimens of *N.tenuipes*.

### ﻿Genetic comparisons

**Figure 2.**
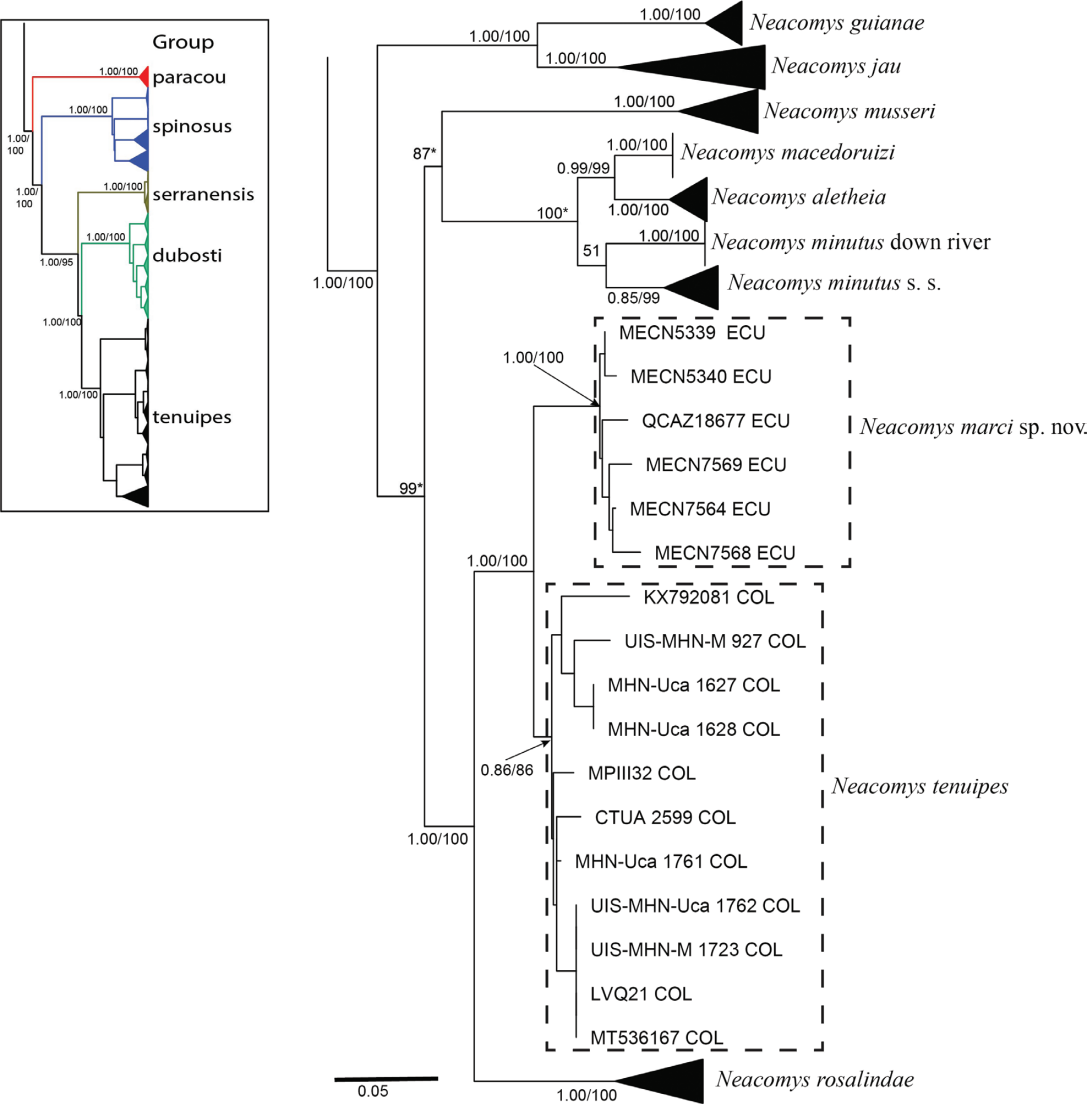
On the left is the Maximum Likelihood phylogenetic tree of the genus *Neacomys* based on the mitochondrial Cytb gene. On the right, Maximum Likelihood phylogenetic tree, extension of the “*tenuipes*” group. The numbers above the nodes represent the values of posterior (left) and bootstrap (right) probabilities. The * represents differences in the relationship found in the Bayesian Inference tree.

*Neacomys* was recovered as a monophyletic group (BS: 100/ PP: 1.00; Fig. 2), with five nested subclades mostly congruent with the species groups mentioned by other authors (Hurtado and Pacheco 2017; Semedo et al. 2020; Brito et al. 2021a; Colmenares-Pinzon 2021). The inclusion of the Serrano Spiny Mouse, *N.serranensis*, and the Golden-belly Spiny Mouse *N.auriventer* to our phylogenetic analyses demonstrated that these morphologically and ecologically similar species are closely related, thus forming the novel “*serranensis*” group (Fig. 2). The ML analysis (Fig. 2) obtained the following relationship for the groups: “*paracou*” + [“*spinosus*” + {“*serranensis*” + (“*dubosti*” + “*tenuipes*”)}]. Relationships between species groups and between species in these groups were mostly consistent with previous phylogenetic hypotheses (Colmenares-Pinzon 2021; Brito et al. 2021a). The samples identified as *Neacomystenuipes* from Ecuador and Colombia were grouped into two sister clades (Fig. 2), each clade presents high support Ecuador (100/1.00) and Colombia (86/0.86).

Calculated divergence between these two lineages was 4.35%±1.18% (Table 3), a value that is comparable with the divergences between well discrete species such as *N.marajoara* and *N.xingu* (4.0%), *N.macedoruizi* and *N.aletheia* (4.8%), *N.vossi* and *N.xingu* (5.4%), *N.marajoara* and *N.vossi* (5.5%), and *N.macedoruizi* and *N.minutus* (5.6%).

**Table 3.** Uncorrected genetic distances of species of the genus *Neacomys* formally described (21 species). We calculated the genetic distance based on the Cytochrome b gene. The values to the right of the diagonal are the standard deviation.

		1	2	3	4	5	6	7	8	9	10	11	12	13	14	15	16	17	18	19	20	21
1	*N.serranensis*		1.40	1.42	1.43	1.32	1.53	1.42	1.23	1.10	1.34	1.48	1.72	1.64	1.53	1.53	1.52	1.35	1.40	1.33	1.59	1.64
2	*N.tenuipes*	15.41		1.31	1.23	1.00	1.32	1.27	1.39	1.35	1.18	1.12	1.44	1.27	1.42	1.18	1.18	1.19	0.68	1.49	1.39	1.27
3	*N.amoenus*	14.25	13.80		0.47	1.50	1.74	1.46	1.35	1.31	1.56	1.07	1.11	1.25	1.41	1.40	1.40	1.43	1.30	1.42	1.27	1.32
4	*N.carceleni*	14.84	13.33	3.36		1.42	1.50	1.49	1.29	1.34	1.40	0.99	1.03	1.24	1.33	1.30	1.21	1.15	1.25	1.48	1.30	1.33
5	*N.rosalindae*	15.48	7.52	15.74	15.19		1.21	1.37	1.25	1.28	1.21	1.24	1.54	1.42	1.48	1.30	1.29	1.07	1.01	1.34	1.41	1.35
6	*N.aletheia*	16.16	11.10	16.19	16.14	11.21		1.60	1.44	1.67	1.50	1.42	1.69	1.46	1.45	1.62	1.06	0.83	1.32	1.57	1.63	1.58
7	*N.musseri*	16.14	11.95	14.77	16.16	12.36	14.21		1.41	1.52	1.40	1.29	1.69	1.38	1.31	1.47	1.44	1.36	1.36	1.56	1.41	1.43
8	*N.paracou*	15.18	15.59	16.26	15.88	15.50	16.88	17.32		1.39	1.49	1.33	1.52	1.22	1.31	1.41	1.40	1.33	1.59	1.43	1.53	1.32
9	*N.dubosti*	15.15	13.42	13.56	14.53	13.23	16.82	14.86	17.42		1.31	1.56	1.75	1.20	1.32	1.45	1.40	1.33	1.48	1.20	1.33	1.26
10	*N.guianae*	19.26	11.42	16.15	16.66	12.63	13.46	15.29	17.03	15.29		1.42	1.76	1.31	1.37	1.03	1.57	1.62	1.39	1.35	1.44	1.33
11	*N.vargasllosai*	15.29	13.08	9.56	8.94	13.85	15.12	13.68	14.49	14.99	15.79		1.09	1.31	1.42	1.23	1.38	1.34	1.23	1.65	1.45	1.36
12	*N.spinosus*	15.15	13.59	8.51	8.32	13.48	15.23	14.43	15.27	15.55	16.80	8.55		1.54	1.67	1.56	1.51	1.55	1.58	1.57	1.59	1.49
13	*N.marajoara*	15.48	12.68	13.44	13.78	13.92	13.93	15.42	15.11	10.23	15.00	14.21	15.71		0.77	1.46	1.44	1.24	1.24	1.47	1.12	0.88
14	*N.xingu*	15.39	13.10	14.27	14.23	13.99	13.09	14.60	16.63	11.11	15.21	14.54	16.61	4.17		1.51	1.45	1.23	1.39	1.57	1.08	0.87
15	*N.jau*	18.22	12.97	17.13	16.33	13.19	14.80	15.32	15.10	13.91	8.91	15.01	15.36	14.66	15.39		1.46	1.48	1.40	1.50	1.46	1.37
16	*N.minutus*	16.30	11.10	15.38	15.69	12.59	7.66	12.60	16.32	14.63	13.90	14.51	14.96	14.67	14.01	14.71		0.81	1.18	1.34	1.30	1.47
17	*N.macedoruizi*	15.28	10.43	15.22	14.65	11.21	4.91	13.87	15.80	14.23	13.50	14.84	14.73	13.80	12.61	14.60	5.68		1.19	1.44	1.36	1.36
18	*N.marci* sp. nov.	15.26	**4.35**	14.86	14.21	8.43	11.66	12.84	15.43	14.31	12.38	14.27	14.09	13.46	14.19	13.37	10.55	10.87		1.65	1.41	1.31
19	*N.auriventer*	**12.55**	14.95	12.99	14.34	14.30	15.85	15.21	16.00	14.42	17.00	16.14	13.57	15.48	14.80	15.04	14.29	14.89	15.86		1.41	1.57
20	*N.elieceri*	16.26	14.52	14.36	14.02	14.72	15.64	15.28	17.56	11.95	16.06	15.03	16.08	9.64	7.70	13.84	13.56	15.03	15.01	14.30		1.02
21	*N.vossi*	15.76	12.80	13.86	14.14	14.16	14.63	16.27	16.64	11.16	15.10	13.65	15.73	5.76	5.63	13.62	15.38	14.58	13.75	14.76	8.23	

These results, along with those from the morphological qualitative and quantitative comparisons constitute strong evidence of cryptic diversity within *N.tenuipes* and that therefore, recently collected specimens from northwestern Ecuador (Chocó Biogeographic region) represent a species clearly distinct from Colombia. Accordingly, this new species is described as follows.

### ﻿Taxonomy

**Family Cricetidae Fisher, 1867**

**Subfamily Sigmodontinae Wagner, 1843**

**Tribe Oryzomyini Vorontsov, 1959**

#### ﻿Genus Neacomys

Taxon classificationAnimaliaRodentiaCricetidae

Thomas, 1959

29E80340-7831-532E-BC42-8FB962835ED1

##### Type species.

*Neacomystenuipes* Thomas, 1900: holotype UKNHM 1899.10.3.74; type locality “Guaquimay, near Bogota,” Cundinamarca, Colombia.

#### ﻿ Neacomys marci

Taxon classificationAnimaliaRodentiaCricetidae

Brito & Tinoco sp. nov.

8B97D15E-0C7D-5F20-913F-A0DFE2C96EE9

https://zoobank.org/79122A9B-991F-4B46-AEDE-BD740EEB4EB4

Neacomys tenuipes : Brito et al. 2021a; Curay et al. 2022 (non Neacomystenuipes Thomas, 1900).

##### Holotype.

MECN 6232 (field number JBM 2307), an adult female captured on 18 November 2020, by J. Brito, J. Curay and K. Cuji, preserved as dry skin, skull, and skeleton, with muscle and liver sample preserved in 95% ethanol.

##### Measurements of holotype (in mm).

HBL 70; TL 84; HF 20; E 13; w 14.5; CIL 18.5; LIF 2.9; BIF 1.4; LD 5.3; LM 2.5; AW 4; BPB 2.1; LR 6.4; LN 8.2; RW-2 4; LIB 4.2; OL 6.6; BZP 1.7; ZB 11; BB 10.4; OCB 5; BOL 3.1; CD 8.1; BM1 0.8. All measurements of the type series are listed in Table 4.

**Table 4.** Summary of morphometric measurements of all specimens in mm. Species names are accompanied by number of analyzed individuals between parentheses. Mean and standard deviation values are shown between parentheses. Abbreviations of characters are detailed in the text.

	*N.marci* sp. nov. (*n* = 23)	N.cf.pictus (*n* = 2)	*N.rosalindae* (*n* = 62)*	*N.tenuipes* (*n* = 21)**
HBL	62–85(71.39±4.74)	79–90(84.5±7.78)	62–99(75.43±6.52)	70–97(82.21±6.68)
TL	50–88(79.66±9.59)	76–89(82.5±9.19)	61.5–87(76.56±5.34)	80–108(97.44±7.02)
HF	18–22(20.26±1.25)	22–23(22.5±0.71)	17–23(19.86±1.07)	20–23.3(22.16±0.85)
Ear	10–16(12.96±1.49)	14	11–20(13.74±1.45)	13–17(14.94±1.19)
CIL	17.5–18.9(18.31±0.36)	21.48–22.52(22±0.74)	16.8–19.5(18.17±0.56)	17.9–19.9(18.96±0.64)
LIF	2.1–3.1(2.83±0.23)	3.16–3.5(3.33±0.24)	1.9–3.5(2.83±0.23)	2.6–3.7(3.12±0.27)
BIF	1.3–1.6(1.5±0.09)	1.5–1.71(1.6±0.15)	1.2–1.6(1.39±0.09)	1.4–1.9(1.57±0.11)
LD	4.9–5.6(5.25±0.13)	6.32–6.5(6.41±0.13)	4.6–5.9(5.2±0.25)	4.8–5.8(5.4±0.28)
LM	2.3–2.7(2.55±0.1)	3.2–3.24(3.22±0.03)	2.4–2.8(2.61±0.09)	2.6–2.9(2.81±0.1)
AW	3.8–4.3(4.04±0.12)	4.75–4.87(4.81±0.09)	3.5–4.1(3.81±0.17)	3.9–4.4(4.14±0.12)
BPB	2.1–2.5(2.25±0.12)	2.76–2.77(2.76±0.01)	1.9–2.8(2.35±0.17)	2.2–2.5(2.3±0.1)
LR	5.1–6.7(6.2±0.39)	5.88–7.58(6.73±1.2)	5.9–7.4(6.61±0.29)	6.3–7.5(6.98±0.37)
LN	7.3–8.6(8.04±0.35)	8.81–9.22(9.02±0.29)	6.9–8.9(8.08±0.34)	7.3–9.2(8.42±0.53)
RW-2	3.8–4.3(4.06±0.13)	4.83–5.05(4.94±0.16)	3.4–4.5(3.96±0.21)	3.2–3.7(3.43±0.14)
LIB	4.2–4.6(4.41±0.13)	4.68–5.05(4.86±0.26)	3.7–4.6(4.16±0.22)	4.1–4.7(4.38±0.13)
OL	6.2–8.1(6.8±0.36)	8.26–8.83(8.54±0.4)	6.5–7.7(7±0.25)	3.1–4.1(3.54±0.26)
BZP	1.6–2.1(1.83±0.12)	2.3–2.5(2.38±0.13)	1.4–2.1(1.79±0.13)	1.6–2.1(1.87±0.12)
ZB	10.4–11.3(10.94±0.24)	12.6–13.1(12.87±0.33)	9.8–11.5(10.67±0.39)	10.2–11.9(11.21±0.41)
BB	10.2–10.8(10.54±0.17)	10.9–11.3(11.13±0.26)	9.2–10.7(10.09±0.3)	9.9–11.2(10.51±0.3)
OCB	5.03–5.58(5.33±0.15)	6–6.1(6.05±0.07)	4.7–5.5(5.12±0.17)	4.9–5.9(5.36±0.26)
BOL	2.7–3.17(2.94±0.13)	3.56–3.83(3.7±0.19)	2.4–3.3(2.88±0.17)	2.7–3.3(2.98±0.15)
CD	7.54–8.63(7.9±0.24)	8.64–8.75(8.7±0.08)	7.3–8.5(7.82±0.22)	7.4–8.3(7.84±0.23)

* = Sánchez–Vendizú et al. (2018); ** = Caccavo and Weksler (2021).

##### Type locality.

Reserva Dracula, Estación Fisher, Parroquia Chical, Cantón Tulcán, Provincia Carchi, Ecuador, Coordinates: 1.006667, -78.2247; WGS84 taken by GPS at the site of collection; elevation 1,067 m.

##### Paratypes

**(*n* = 38).**MECN 6230, adult male, and MECN 6233, adult female, preserved as dry skin and cleaned skull, collected in Provincia de Carchi, Reserva Dracula, Estación Fisher (1.006667, -78.2247, 1,067 m.) on 18 November 2020, by J. Brito, J. Curay and K. Cuji. MECN 6231, adult male, preserved as dry skin and cleaned skull, collected in Provincia de Carchi, Reserva Dracula, Estación Fisher (1.006667, -78.2247, 1,067 m.) on 20 November 2020, by J. Brito, J. Curay and K. Cuji. MECN 6238, MECN 6239, MECN 6240, MECN 6241, adult males, and MECN 6237, MECN 6242, adult females, preserved in 75% ethanol, collected in Provincia de Carchi, Reserva Dracula, Estación Fisher (1.006667, -78.2247, 1,067 m.) on 21 November 2020, by J. Brito, J. Curay and K. Cuji. MECN 6479, adult male, preserved in 75% ethanol, collected in Provincia de Carchi, Reserva Dracula, Estación Fisher (1.006667, -78.2247, 1,067 m.) on 30 March 2021, by J. Brito. J. Castro, Z. Villacís and J. Guaya. MECN 5339, MECN 5340, MECN 5374, MECN 5375, adult males, preserved as cleaned skulls and carcasses in ethanol, MECN 5370, MECN 5373, adult males, preserved in ethanol, MECN 5372, adult female, preserved as cleaned skull and carcass in ethanol, collected in Provincia de Carchi, Reserva Drácula, Peñas Blancas (0. 973758, -78.210173, 1,290 m) on 27 November 2016, by J. Brito, J. Robayo and H. Yela. MECN 5357, adult male, preserved as cleaned skull and carcass in ethanol, collected in Provincia de Carchi, Reserva Dracula, Pailón (0.992406, -78.237714, 1,270 m) on 29 November 2016, by J. Brito, J. Robayo and H. Yela. MECN 6013, juvenile male, preserved as cleaned skull and carcass in ethanol, collected in Provincia de Carchi, Reserva Dracula, Pailón (0.992406, -78.237714, 1,270 m) on 7 November 2017, by J. Brito, J. Curay and R. Vargas. MECN 5919, adult male, preserved as cleaned skull and carcass in ethanol, collected in Provincia de Carchi, Reserva Dracula, Pailón Alto (0.97415, -78.2176, 1,630 m) on 28 March 2018, by J. R. Vargas and M. Esparza. MECN 5904, adult male, preserved as dry skin and cleaned skull, MECN 6014, adult male, MECN 6015, juvenile male, MECN 6016, adult female, preserved in ethanol, collected in Peñas Blancas on 7 November 2017, by J. Brito, J. Curay and R. Vargas. MECN 6570, adult male, preserved as cleaned skull and carcass in ethanol, collected in Provincia de Imbabura, Parroquia Lita, Aguinaga (0.78125, -78.318113, 1,400 m) on 1 March 2020, by S. Erazo and D. Mantilla. MECN 6271, adult male, preserved in ethanol, collected in Provincia de Imbabura, Reserva Río Manduriacu (0.309547, -78.856631, 1,200 m) on 12 September 2019, by R. Peña. MECN 6766, adult female, preserved as skin dry, skull and skeleton, collected in Pichincha, Reserva Chontaloma (0.18138, -78.90516, 630 m) on 15 March 2021, by S. Pozo and C. López. MECN 7125, juvenile female, preserved as cleaned skull and carcass in ethanol, collected in Pichincha, El Progreso (0.164608, -78.767156, 1,140 m) on 21 September 2021, by R. Garcia. QCAZ 18677, adult male, preserved as dry skin and clean skull / jaw, collected in Pichincha, Reserva Mashpi (0.166600, -78.880000, 900 m) on 26 September 2019, by J. Cook and J. Dunnum. MECN 7563, MECN 7568, adult females, and MECN 7569 adult male, preserved as dry skins and cleaned skulls, MECN 7572, adult female, and MECN 7560, 7561, 7565, 7570, 7573 adult males, preserved as cleaned skull and carcass in ethanol, collected in Provincia de Esmeraldas, Reserva Canandé, Gualpí de los Cayapas (0.56479, -79.06104, 450 m) on 14–16 October 2022, by J. Brito, J. Guaya, and A. Aguilar.

##### Etymology.

Named in honor of Marc Hoogeslag of Amsterdam, the Netherlands. He was co-founder and leader of the innovative Land Acquisition Fund of the International Union for the Conservation of Nature - Netherlands, which helps local groups throughout the world to establish new ecological reserves and conserve endangered species. Fundacion EcoMinga’s Reserva Manduriacu, the habitat of this new species, is one of the many reserves which have benefited from Marc’s program. The species epithet is formed from the surname “Marc” taken as a noun in the genitive case, adding the Latin suffix “i” (ICZN 31.1.2).

##### Diagnosis.

A species of *Neacomys* with the following combination of characters: small size (head-body length 65–85 mm), long tail (69–126% longer than head and body length), belly fur pale buff but with gray based hairs, white throat, long nasals (which extend well beyond the plane of the lacrimal), condylar process higher than coronoid process, M1 anterocone divided, M1 with broad protoflexus; m1–m3 with wide hypoflexids.

##### Morphological description.

**Figure 3.**
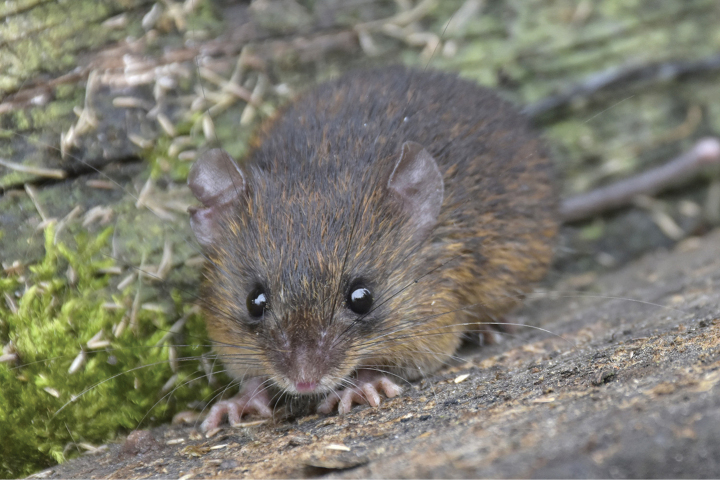
Live specimen of *Neacomysmarci* sp. nov. in its natural habitat (MECN 6230, Estación Fisher, Ecuador). Please note the color of a living animal.

The following description was based on all specimens available. *Neacomysmarci* sp. nov. is a spiny mouse of small size (head and body length 65–85 mm). The dorsal pelage is dark brown (Fig. 3); soft hairs are mixed with spines; on average dorsal hairs are 9–10 mm in length. The soft hair is tricolor, with a light brown band at the base, an orange band in the middle and a black apical band. The posterior mystacial vibrissae are thick and long (34 mm), surpassing the auricular pinnae when ad pressed back; two superciliary vibrissae, the longest measuring 39 mm, extending to the middle of the dorsum. One medium-sized genal vibrissae (32 mm) are also present, which are more slender than the mystacial vibrissae. The ears are large (12–16 mm) and oval in outline. Although the ears seem to be naked, they are covered with short black fringe of hair. The base of the internal ears is yellowish cream and the edges are dark, the hairs are yellowish and medium in size. A small pale orange postauricular patch is present.

**Figure 4.**
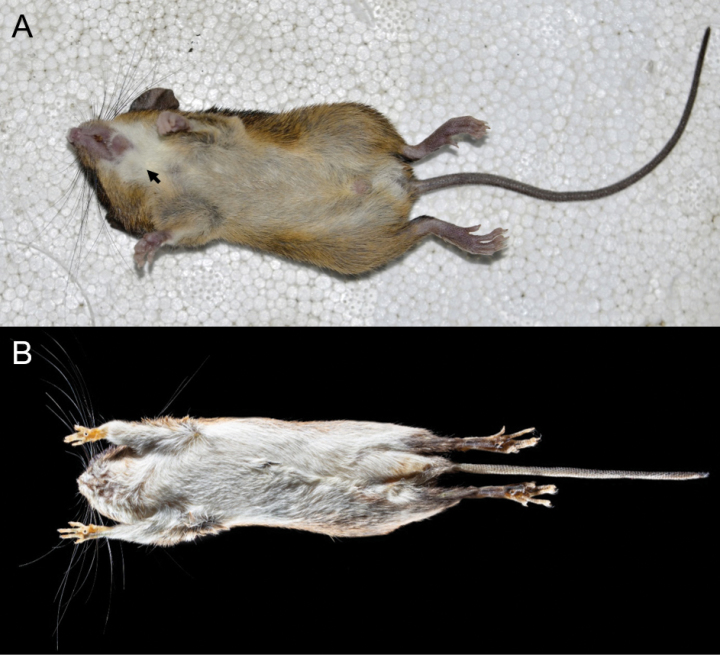
Ventral views of the skin of **A***Neacomysmarci* sp. nov. (MECN 6232, holotype; Estación Fisher, Ecuador), and **B***Neacomystenuipes* (UIS-MHN-M 1723; Finca La Bufalera, Colombia). Note the white-furred throat in *N.marci* sp. nov. (arrowed).

The pelage on the throat is white (Fig. 4A) and extends up to the corners of the mouth. The ventral pelage is pale buff but with gray base, and the hairs are on average 3.0–3.5 mm in length at the middle of the belly. The tail is uniformly dark, slender, and long (69–126% longer than head and body length). It is covered with rectangular scales (13 or 14 rows/cm near the base), with three dark brown hispid hairs emerging from the base of each scale, not longer than 1.5–2 scale rows. The hairs of the terminal portion of the tail form a small tuft (< 3 mm). Females have eight mammae arranged in pectoral, thoracic, abdominal, and inguinal pairs.

The manus is slender and short. The first digit is reduced with a long and wide claw. The other claws are short and curved. Ungual tufts are white and extend beyond the claw ends. The dorsal surface with evident brown scales; each scale has three dark brown hairs and sometimes the central hair is the longest. Long carpal vibrissae can reach the claw of digit V. The digits are relatively large; digit I is substantially shorter than digit II; digit II is shorter than digit III; digit III is slightly larger than digit IV; digit IV is larger than digit V.

**Figure 5.**
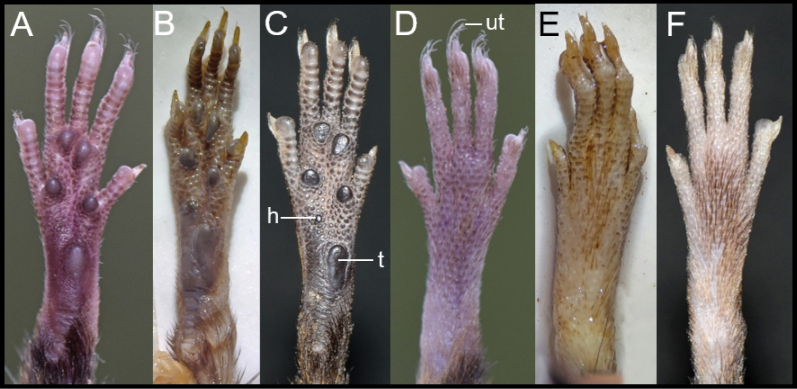
Ventral (**A–C**) and dorsal (**D–F**) views of the hind foot of *Neacomysmarci* sp. nov. (**A, D**MECN 6232, holotype; Estación Fisher, Ecuador), *N.tenuipes* (**B, E**MHN-UCa-M 4019, Colombia), and *N.rosalindae* (**C, F**MECN 5824; Cordillera de Kutukú, Ecuador). Figures are not to scale to facilitate comparisons. Abbreviations: h = hypothenar, t = thenar, ut = ungual tufts.

Hind feet are long and slender (18–22 mm); the ungual tufts are white, abundant and extend well beyond the edge of the claws (Fig. 5A, D). Their dorsal surface has a small metatarsal patch, with brown scales (Fig. 5D); each scale has three dark brown hairs. Large number of granules covers most of the plantar surface, including the spaces between the pads and reaching the anterior border of the thenar pad. The four interdigital pads are elevated and similar in size; pads II and III are separated by a small interspace, while pads II and IV are separated by an interspace of similar size than pad I (Fig. 5A). The hypothenar pad is very small or absent, while the thenar pad is well developed, large and elevated anteriorly. Digits are relatively short; digit I reaches the base of digit II; digit II is slightly shorter than digit III; digit III is slightly larger than digit IV; digit IV is larger than digit V; digit V reaches halfway of the first phalanx of digit IV (Fig. 5A, D); claws are short, recurved and basally opened.

**Figure 6.**
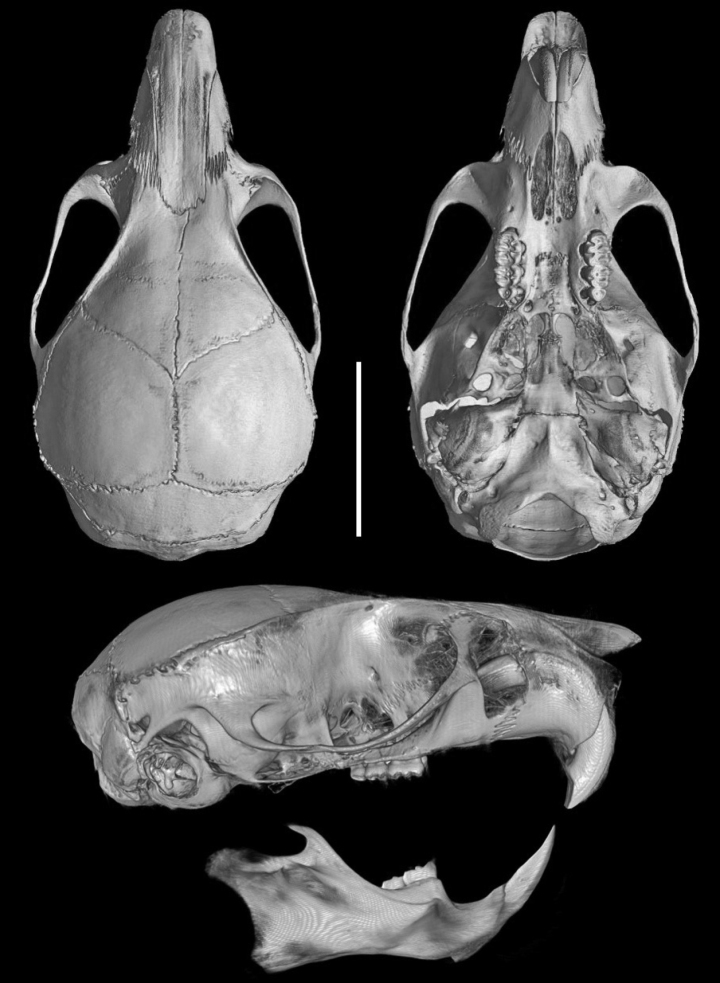
Three-dimensional reconstruction of the skull of *Neacomysmarci* sp. nov., based on micro-CT data of the holotype (MECN 6232; Estación Fisher, Ecuador): cranium in dorsal, ventral, and lateral view, and left hemimandible in labial view. Scale bar: 5 mm.

**Figure 7.**
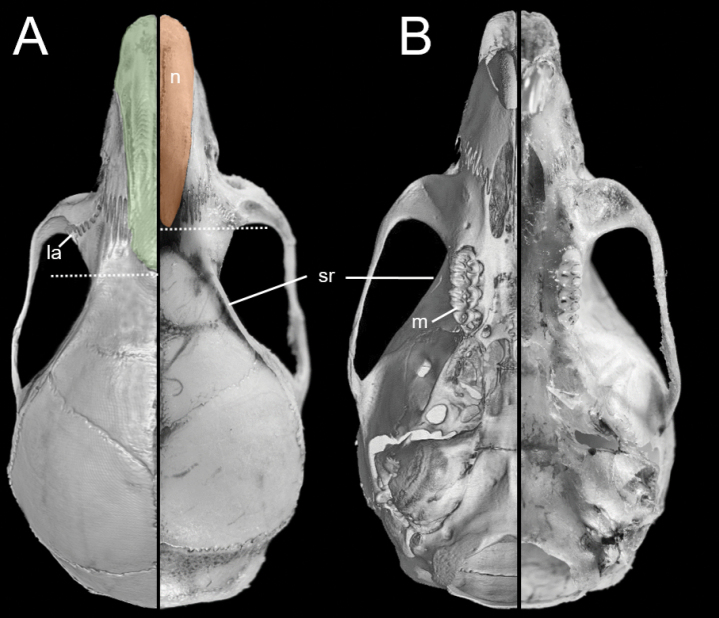
Selected aspects of qualitative anatomy contrasted in the crania (dorsal view = **A**, ventral view = **B**) based on data of *Neacomysmarci* sp. nov. (left; MECN 6232, holotype; Estación Fisher, Ecuador) and *Neacomystenuipes* (right; UIS-MHN-M 1723; Finca La Bufalera, Colombia). The figure portrays differences between the characteristics of these species as follows: *N.marci* sp. nov. has the longest nasal (n) extending well beyond the plane of the lacrimals (la), larger molars (m), and a low sagittal ridge (sr). Figures are not to scale to facilitate comparisons.

**Figure 8.**
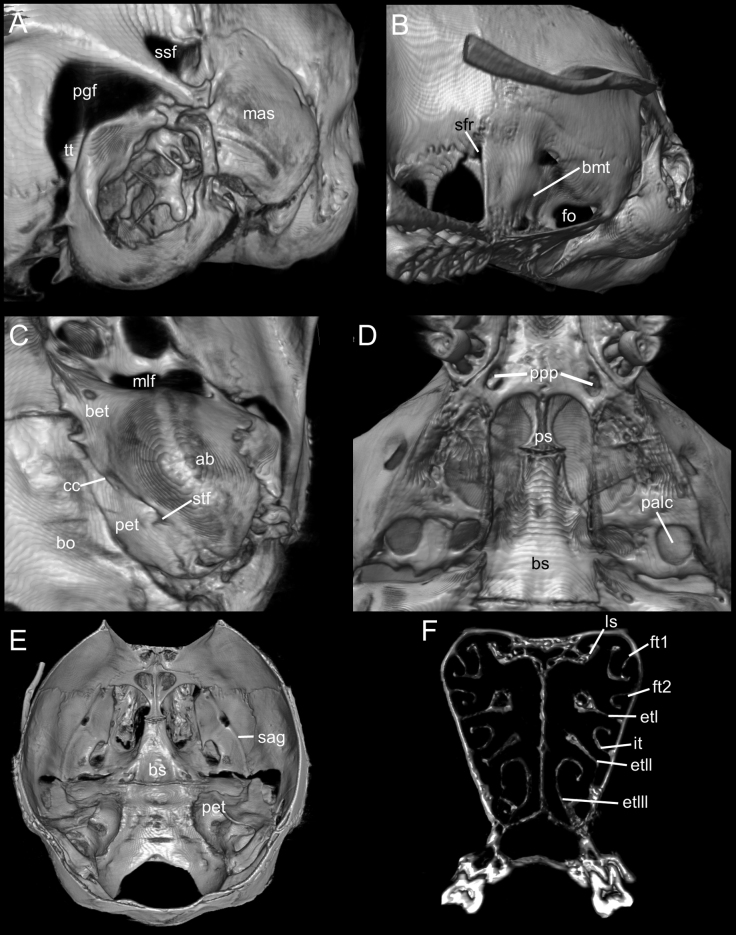
**A** selected anatomical features of the skull of *Neacomysmarci* sp. nov. based on the holotype (MECN 6232; Estación Fisher, Ecuador): posterior portion of the skull in lateral view **B** lateral view of alisphenoid bone region **C** right auditory region in ventral view **D** ventral view of basicranial region **E** dorsal view (roofing bones of braincase removed) of basicranial region **F** cross-section of the cranium. Abbreviations: ab, auditory bulla; bet, bony Eustachian tube; bmt, buccinators-masticatory trough; bo, basioccipital; bs, basisphenoid; cc, carotid canal; etI, ethmoturbinal I; etII, ethmoturbinal II; etIII, ethmoturbinal III; fo, foramen ovale; ft1, frontoturbinal 1; ft2, frontoturbinal 2; it, interturbinal; ls, lamina semicircularis; mas, mastoid capsule; mlf, middle lacerate foramen; palc, posterior opening of the alisphenoid canal; pet, petrosal; pgf, postglenoid foramen; ppp, posterior palatal pits; ps, presphenoid; sag, squamosal alisphenoid groove; sfr, sphenofrontal foramen; stf, stapedial foramen; ssf, subsquamosal fenestra; tt, tegmen tympani; Pictures are three-dimensional reconstructions based on micro-CT data.

The cranium is moderately large for the genus (average CIL = 18.2 mm) with the braincase showing a convex profile (Fig. 6). The dorsal profile of the cranial roof is flat from the nasals to the middle of the frontals, then rises at the back of the frontals and slopes gently down the parietals toward the occiput; the rostrum is long and slender; premaxillae are slightly shorter than nasals, not extending anteriorly beyond incisors, without forming a rostral tube; gnathic process is very small; the suture between the nasal bones and the premaxillary reaches the root of the zygomatic bone; the nasal bone is wide at the base and gradually widens forward (Fig. 7); the interorbital region is narrow; the supraorbital edges are small and sharp; the zygomatic notches are shallow and wide while seen from above; in the olfactory sagittal plane are two frontoturbinals, one interturbinal and three ethmoturbinals present (Fig. 8F); the lachrymal is small, with contact in equal proportions with the frontal and maxillary; the post-nasal depression is shallow; the fronto-parietal suture is V-shaped; the parietal is restricted to the dorsal portion of the skull; the braincase is rounded and inflated. A gnatic process is not developed; the zygomatic plate is wide and excavated (> M1 length) and slightly inclined backward; the zygomatic arch slender and without a jugal; a squamosal-alisphenoid groove is visible through the translucent braincase (Fig. 8B, E), with a perforation where it crosses the depression for the masticatory nerve; the stapedial foramen is present and small, the carotid canal is small, and the petrotympanic fissure is expressed (Figs 8C); the cephalic arterial supply is primitive (pattern 1 of Voss 1988); the alisphenoid strut is absent; an anterior opening of the alisphenoid canal is absent; the postglenoid foramen is large; the subsquamosal fenestra is small and the hamular process of the squamosal is long; a small tegmen tympani is present (Fig. 8A); there is no contact between the anterodorsal edge of the ectotympanic and the mastoid tubercle, which leads to an opened ectotympanic ring (Fig. 8A); the orbicular apophysis of the malleus is wide and elongate (oval in shape), with its longitudinal axis inclined towards the manubrium; mastoid bears no dorsolateral fenestra; the paraoccipital process is short.

The Hill foramen is tiny; the incisive foramina are short, ending well anterior to the M1s anterior faces; the capsular process of the premaxillary is well developed; the palate is wide and long with the anterior border of the mesopterygoid fossa not reaching M3s posterior faces; the palatal foramina are small; the posterolateral pits are long and paired, and located parallel to the anterior part of the mesopterygoid fossa; the mesopterygoid fossa is broad as the parapterygoid plates, with the anterior margin U-shaped (Fig. 8D); the shape of the pterygoid plate is not expanded, and has straight margin; the sphenopalatine vacuities are elongated and narrow, occupying the posterior part of the presphenoid area; the presphenoid is wide (Fig. 8D); the auditory bullae are small and flask-shaped; the Eustachian tube is short, wide and gradually constricted; the petrosals are well-exposed; the anterior bullae process is in contact with the posterior margin of the pterygoid plate (Fig. 8C); the basioccipital depressions are deep, forming an recognizable crest; the anterior border of the foramen magnum is narrow, with a conspicuous notch.

The mandible with masseteric crest in line with procingulum of m1; the coronoid process is small, slender, and bended backwards; the sigmoid notch is oval; the condylar process is large and robust; the capsular process is forming a rounded elevation that lies below the coronoid process; the angular notch is shallow, and the angular process is blunt.

**Figure 9.**
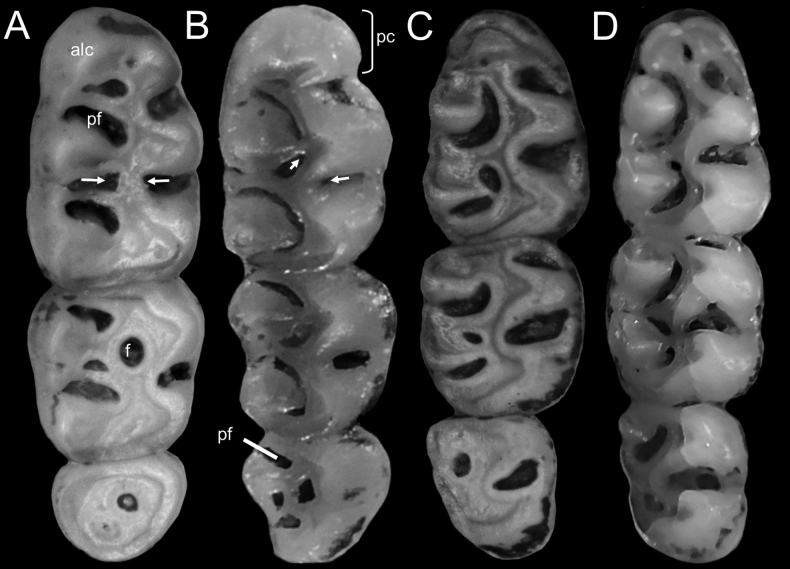
**A, B** occlusal view of the right upper, and **C, D** right lower tooth row of: (**A, C**) *Neacomysmarci* sp. nov. (MECN 6232, holotype; Estación Fisher, Ecuador) and (**B, D**) *N.tenuipes* (**B**UIS-MHN-M 1723; Finca La Bufalera, Bolívar, Colombia, and **D**MHN-UCa-M 3647; Acevedo, Huila, Colombia). Abbreviations: alc, anterolabial conule; pf, paraflexus; f, mesofosette; pc, procingulum.The arrows indicate the direction of the mesoflexus with the hypoflexus.

The incisors are opistodont, without grooves, and with yellowish enamel; the molars are brachydont and terraced (Fig. 9A); the main cusps of the upper and lower molars are opposed. The M1 is rounded in outline; the procingulum is narrower than the rest of the molar, with a rounded anteromedian fossette present; anterocone divided; the protoflexus is broad; the mesoflexus is small; the metaflexus is large and wide; the posteroloph is small. The M2 with indistinct protoflexus; the anteroloph is small; the mesoflexus is short and wide; the mesoloph is short; the mesofosette is rounded (Fig. 9A); the posteroloph is similar to M1. The M3 has a small paraflexus and indistinct hypoflexus. The upper molars have three roots each. The m1 is rectangular in outline; the procingulum is not divided into labial and lingual conulids; the protoflexid is short and wide; the hypoflexid is wide; the mesoflexid is large and wide; the mesolophid is large; the posteroflexid is short and broad; the mesofosette is large. The m2 is square in outline; the protoflexid is large and narrow; the hypoflexid is wide and inclined with direction towards the posteroflexid; the mesoflexid is short and wide; the mesolophid is short and wide; the mesofosette is very small. The m3 is anteriorly-posteriorly compressed, having a wide hypoflexid and a small anterolabial cingulum. The lower molars have two roots each.

The tuberculum of the first rib articulates with the transverse processes of the seventh cervical and the first thoracic vertebrae; the second thoracic vertebra has a differentially elongated neural spine; 19 thoracicolumbar vertebrae, the 16^th^ with moderately developed anapophyses; four sacrals; 33 or 34 caudals, with complete hemal arches in the second, third and fourth; 12 ribs.

**Figure 10.**
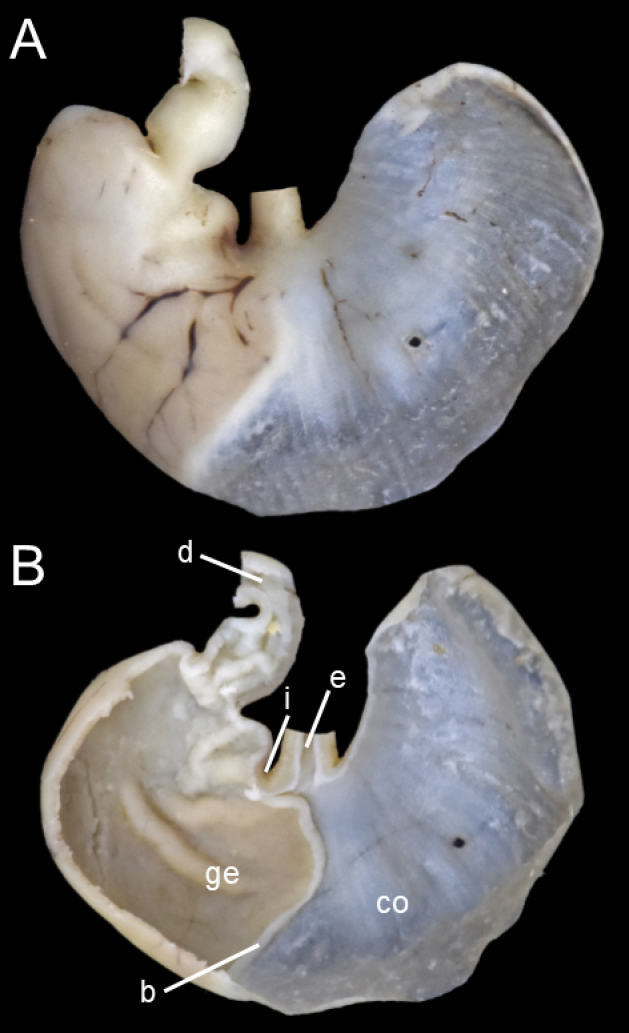
Stomach of *Neacomysmarci* sp. nov. (MECN 7568; Reserva Río Canandé, Ecuador) **A** dorsal view **B** ventral view. Abbreviations: b, bordering fold; d, duodenum; co, cornified epithelium; ge, glandular epithelium; i, incisura angularis; e, esophagus.

The gall bladder is absent. The stomach is unilocular and hemiglandular; the cornified epithelium lines the corpus, while the glandular epithelium occupies the antrum and is slightly extended to the left of the esophageal opening; the bordering fold is notorious for being thick and long, surpassing the left level of the incisura angularis; the incisura angularis is moderately deep and the plica angularis is well expressed with a well-developed pars pyloricus (Fig. 10).

##### Comparisons with similar species.

*Neacomysmarci* sp. nov. differs from its sister species *N.tenuipes* mainly in ventral coloration, *N.marci* sp. nov. is pale buff with white throat, while *N.tenuipes* is completely white to pale orange (Fig. 4). Additionally, *N.marci* sp. nov. has a slight bicolor at the base tail, while *N.tenuipes* has a clear bicolor at the base. The condylar process in *N.marci* sp. nov. is higher than the coronoid process, while in *N.tenuipes* most are lower than the coronoid process, some are equal to the coronoid process. At the molar level, *N.marci* sp. nov. has a narrow anterocone of M1, while in *N.tenuipes* it is wide (Fig. 4). In *N.marci* sp. nov. the hypoflexus of M3 is indistinct or absent, while in *N.tenuipes* it is present and well evident.

Another species from the Chocó Biogeographic region with which *Neacomysmarci* sp. nov. could be confused is *N.pictus*. Both species have white throats, however *N.marci* sp. nov. has pale buff ventral color and *N.pictus* is faintly plumbeous basally on the belly. *Neacomysmarci* sp. nov. has the interorbital region (in ventral view) with developed ridges, projecting like ledges; whereas *N.tenuipes* it is hidden under the maxilla. The mastoid is ossified in *N.marci* sp. nov. while in *N.tenuipes* it is most perforated. Other comparisons are summarized in Table 5.

**Table 5.** Selected morphological comparisons between *Neacomys* species distributed in the Chocó Biogeographic region. Characters obtained analyzing photos of the holotype and the description supplied by Colmenares-Pinzon (2021) * and Caccavo and Weksler (2021) **.

Characters	*Neacomysmarci* sp. nov. (*n* = 30)	Neacomystenuipes*	Neacomyspictus**
Tail length	most subequal to HBL	most subequal to HBL	subequal to HBL
Tail color	unicolor	bicolor	most bicolor
Hypothenar pad	absent	present	–
Ventral fur color	pale buff, with white throat	completely white to pale orange	faintly plumbeous basally on belly, with throat white
Lacrimal bones	equal contact with frontal and maxillary bones	equal contact with frontal and maxillary or major contact with maxillary bone	major contact with maxillary bone
Post nasal depression	shallow	most deep	shallow
Supraorbital crests	crests developed and inflexed posteriorly	crests developed and inflexed posteriorly	most with crests developed and inflexed posteriorly
Parietal	restricted to the dorsal portion of the skull	restricted to the dorsal portion of the skull	restricted to the dorsal portion of the skull
Mastoid ossification	ossified	ossified or perforated	most perforated
Shape of diastema	flat	flat	with a small bump below the zygomatic plate
Incisive foramina position	distant to M1	close or distant to M1	close to M1
Posterolateral palatal pits	unique or shallow opening	Most unique or shallow opening	unique or shallow opening, or multiple openings
Interorbital region (ventral view)	with developed ridges, projecting like ledges	with developed ridges, projecting like ledges	hidden under the maxilla
Condylar process	higher than coronoid process	lower than coronoid process, some equal to coronoid process	lower than coronoid process, some equal to coronoid process
M1, shape of anterocone	narrow	wide	wide
M1, shape of mesoloph	most straight	most straight	curved
M3, hypoflexus	absent	present	present

##### Distribution.

**Figure 11.**
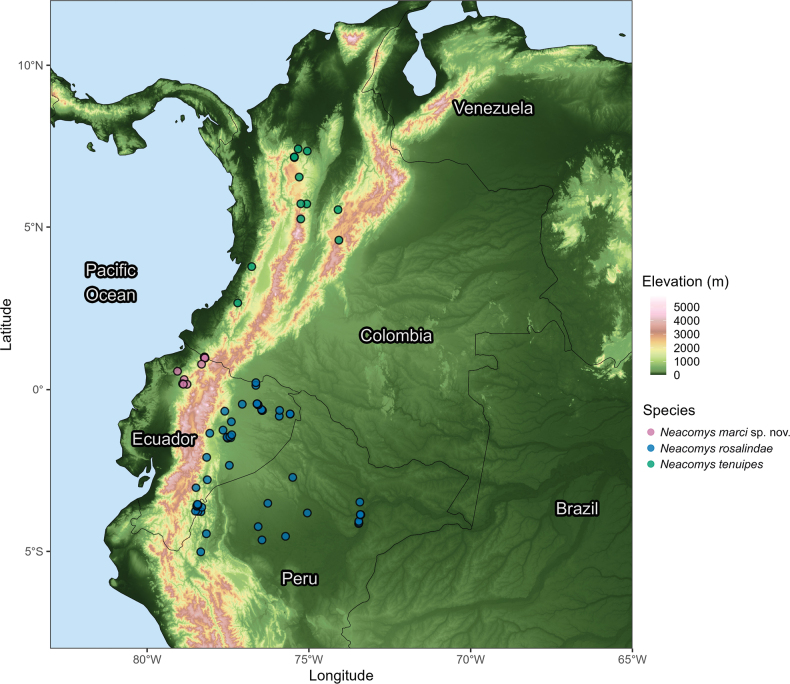
Topographic map of northern South America. Sampling localities of three *Neacomys* species are shown with color codes described in the legend. *Neacomysmarci* sp. nov. localities correspond to the Chocó biogeographic region in northwestern Ecuador (type locality is shown with black circle).

*Neacomysmarci* sp. nov. is known from six localities in the provinces of Carchi, Pichincha, and Esmeraldas, in northwestern Ecuador (Fig. 11).

##### Natural history.

**Figure 12.**
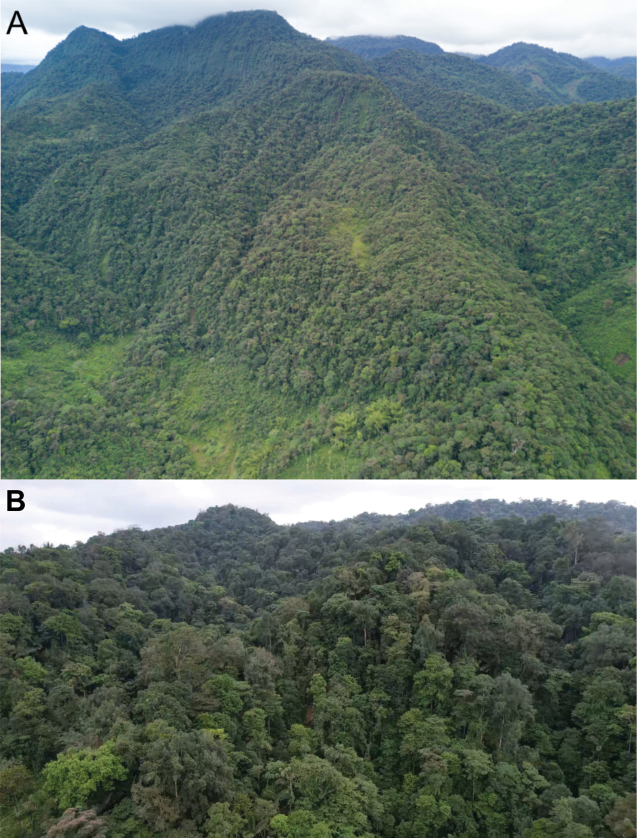
Habitat where specimens of *Neacomysmarci* sp. nov. have been collected in this study **A** Piemontane forest, and **B** Chocó rainforest.

The distributional range of the species is thus far limited to the Chocó Biogeographical region (Myers et al. 2000), where it occupies the lower subtropical and lower montane ecosystems (Ceron et al. 1999), in an altitudinal range from 450 to 1,630 m (Fig. 12). These forests are characterized by having a tree cover of approximately 30 m height. Most of the vegetation belongs to the families Araceae, Melastomataceae, Cyclanthaceae, Bromeliaceae, and to the ferns. Additionally, the following species of rodents and marsupials were recorded as living in sympatry: *Melanomyscaliginosus*, *Mindomyshammondi*, *Oecomys* sp., *Rhipidomyslatimanus*, *Tanyuromysthomasleei*, *Pattonimusmusseri*, *Sigmodontomysalfari*, and *Transandinomysbolivaris*, the heteromyid *Heteromysaustralis*, the marsupials *Chironectesminimus*, *Mamosopscaucae*, and *Marmosaisthmica*, and the squirrel *Microsciurusmimulus*.

## ﻿Discussion

With the description of *Neacomysmarci* sp. nov. the diversity of the genus reaches 24 formally recognized species, of which 14 (60%) have been described in the last five years (Hurtado and Pacheco 2017; Sánchez-Vendizú et al. 2018; Semedo et al. 2020; Brito et al. 2021a; Caccavo and Weksler 2021; Colmenares-Pinzon 2021; Semedo et al. 2021). Such dynamism has not been seen recently in the taxonomy of any other group of oryzomyine rodents and places *Neacomys* as the most diverse group within the tribe, and the third most diverse within the subfamily Sigmodontinae, only comparable to the genus *Oligoryzomys* (Hurtado 2021).

Results presented here confirm that comprehensive revisions of currently recognized species, i.e., by morphological and genetic characterizations, as well as collection of specimens in unexplored regions are fundamental to unveil cryptic diversity in groups of small mammals. Particularly for *Neacomys*, species once considered as homogeneous throughout a wide distribution, such as *N.minutus*, *N.spinosus*, or *N.tenuipes* have been split into multiple taxa. For *N.tenuipes*, Caccavo and Weksler (2021) recognized populations from Venezuela as different (*N.leilae* Caccavo & Weksler, 2021), whereas in this study, we found enough evidence to propose a separation of the populations from northwestern Ecuador into *N.marci* sp. nov. Likewise, other authors have noticed clear differences in other populations from the Chocó region in Colombia, whose genetic characterization is pending, to validate the name *N.pusillus*. On the other hand, it is worth mentioning that other specimens from northwestern Ecuador reviewed here and tentatively identified as N.cf.pictus (QCAZ 708 and MECN 3050), seem to differ notably from this species and from any other species of *Neacomys*. Further collections and the generation of genetic data from this population could result in the recognition of a new species, which ultimately demonstrates that the number of species within the genus will continue to increase.

The rainforests of northwestern Ecuador have both high biodiversity and endemism due to the biogeographic influence of the Chocó and Andes Mountains (Myers et al. 2000). For example, a variety of oryzomyines of the genera *Pattonimus*, *Sigmodontomys*, *Tanyuromys*, *Transandinomys*, and “*Handleyomys*” (Pine et al. 2012; Patton et al. 2015; Brito et al. 2020) are endemic to the Chocó forests. Despite this, our knowledge of the sigmodontine biodiversity of this hotspot is still incomplete.

It is important to mention that after more than two centuries of active research in mastozoology (Tirira 2014), intensive fieldwork was conducted in few places in Ecuador. Examples for those sites in the eastern Andes are Papallacta (Voss 2003), Guandera Biological Reserve (Lee et al. 2015), and Sangay National Park (Brito and Ojala-Barbour 2016), and in the western Andes are Cajas National Park (Barnett 1999), Otonga Reserve (Jarrín 2001), Pululahua Geobotanical Reserve (Curay et al. 2019), Polylepis Forest (Ojala-Barbour et al. 2019), Reserva Dracula (Brito et al. 2020, 2022a), and Lita (Curay et al. 2022). The interest in complementary biodiversity studies has led to the prioritization of intensive field work, using a variety of trapping techniques (e.g., live traps, spring traps, and pitfall traps), and has also triggered revisions of museum specimens. For example, in the last five years, these approaches have led to the description of at least 14 new sigmodontine: five *Chilomys* (see Brito et al, 2022a), three *Thomasomys* (see Brito et al. 2019; Brito et al. 2021b; Lee et al. 2022), one *Tanyuromys* (see Timm et al. 2018), one *Ichthyomys* (Fernández de Córdova et al. 2020), two *Pattonimus* (Brito et al. 2020), one *Neacomys* (Brito et al. 2021a), and one *Mindomys* (Brito et al. 2022b). This burgeoning richness will surely reorganize part of our understanding of Neotropical cricetids. This context highlights the urgency of establishing national and comprehensive inventory and collection programs, including sampling in previously studied areas as well as improving scholarly access to these resources.

## Supplementary Material

XML Treatment for Neacomys

XML Treatment for Neacomys marci

## ﻿Additional information

### Author ORCIDs

Nicolás Tinoco https://orcid.org/0000-0002-2196-1199

Claudia Koch https://orcid.org/0000-0002-7115-2816

Javier E. Colmenares-Pinzón https://orcid.org/0000-0003-2828-5522

Francisco X. Castellanos https://orcid.org/0000-0003-0955-8185

Jorge Brito https://orcid.org/0000-0002-3410-6669

### Data availability

All of the data that support the findings of this study are available in the main text.

## ﻿Appendix 1

Studied specimens belong to the following mammal collections: **CTUA**, Colección Teriológica de la Universidad de Antioquia, Colombia; **USNM**, National Museum of Natural History, Smithsonian Institution, USA; **FMNH**, Field Museum of Natural History, USA; **AMNH**, American Museum of Natural History, USA; **TTU**, Texas Tech University, USA; **IAvH-M**, Instituto de Investigación de Recursos Biológicos Alexander von Humboldt, Bogotá, Colombia; **UIS-MHN-M**, Universidad Industrial de Santander, Santander, Colombia; **MHN-UCa-M**, Universidad de Caldas, Caldas, Colombia; **MECN**, Instituto Nacional de Biodiversidad, Quito, Ecuador; **MEPN**, Museo de la Escuela Politécnica Nacional, Quito, Ecuador; **QCAZ**, Museo Pontificia Universidad Católica del Ecuador, Quito, Ecuador.

*Neacomysmarci* sp. nov. (*n* = 39): Ecuador, Carchi, Reserva Drácula, Estación Fisher: MECN 6232 (holotype), MECN 6230, 6231, 6233, 6237–6242, 6479; Pailón: MECN 5357, 6013; Pailón Alto: MECN 5919; Peñas Blancas: MECN 5339–40, 5370, 5372–75, 5904, 6014–16; Imbabura, Ibarra, Lita, Aguinaga: MECN 6570; Cotacachi, García Moreno, Reserva Río Manduriacu: MECN 6271; Pichincha, Pacto, El Progreso: MECN 7125; Reserva Chonta Loma: MECN 6766; Reserva Masphi: QCAZ 18677; Esmeraldas, Reserva Canandé, Gualpí de los Cayapas: MECN 7560–61, 7563, 7565, 7568–70, 7572–73.

*Neacomystenuipes* (*n* = 60): Colombia, Antioquia, Amalfi: CTUA 2599; Cisneros, 11 Km S and 30 Km E: USNM 499543–45; El Carmen de Viboral, Vereda el Porvenir, Cañón del Río Melcocho; IAvH-M 10291–302; Sonsón, Río Negrito, 15 Km E: FMNH 70130, 70134; Valdivia, 4 Km S, Quebrada de Oro: FMNH 70126; Zaragoza: IAvH-M 2934–35, 2938, 2956, 2959, 2961, 2968, 2980, 3756; 25 Km S, 22 Km W, at la Tirana: USNM 499541, 499546–47, 499549–52, 499554, 499556–59, 499578–79; BOLIVAR, Cantagallo, Vereda Santo Domingo, Finca La Bufalera: UIS-MHN-M 1720, 1723, 1762; Boyacá, Muzo: FMNH 71778; Caldas, Manizales, Ecoparque Los Alcázares: MHN-UCa-M 1761; Samaná, Río Hondo: FMNH 71748, 71762, 71766; Parque Nacional Natural Selva de Florencia, Vereda San Antonio, microcuenca Las Mercedes: MHN-UCa-M 1627–8; Cundinamarca, Medina, Vereda Periquito: IAvH-M 10230; Paime: AMNH 69182; Chocó, Riosucio (IAvH-M 5029–30; Huila, Acevedo, Río Aguas Claras: FMNH 71776; MHN-UCa-M 3647; Santander, Lebrija, Vereda Portugal, Granja El Puente: UIS-MHN-M 927.

*Neacomystenuipespusillus* (*n* = 1): Colombia, from San José, Cauca: AMNH 31695 (holotype).

*Neacomysrosalindae* (*n* = 25): Ecuador, Morona Santiago, Taisha: MECN 476, 468–69; Orellana, Pompeya Sur: MECN 776, 791, 793, 968, 994, 1006; Comunidad Jabalí: MECN 2333; Pastaza, Lorocachi: MEPN 12363, 12371–72, 12387; Bameno: MEPN 12433, 12468, 12470–71, 12474–78; Zamora Chinchipe, Tundayme: MECN 5689–90.

*Neacomyspictus* (*n* = 2): Panama, from Cana, Darien: USNM 178717 (holotype), TTU 39148.

Neacomyscf.pictus (*n* = 2): Ecuador, Esmeraldas, Muisne, Río San Francisco: MECN 3058; Cotopaxi, Reserva La Otonga: QCAZ 808.

## ﻿Appendix 2

**Table A1.** List of specimens included in the phylogenetic analyses. For each terminal species, catalog access numbers and GenBank accessions are provided. An asterisk * denotes holotypes.

Taxa	Cytb	Catalog number or collector	Locality
OUTGROUPS:			
*Microryzomysaltissimus*	EU579502	QCAZ 8353	Ecuador, Azuay
*Microryzomysminutus*	EU258535	MVZ 166666	Perú, Cusco
*Oligoryzomysflavescens*	GU185922	P32	Uruguay, San José
*Oreoryzomysbalneator*	EU579510	AMNH 268144	Perú, Cajamarca
*Oryzomyspalustris*	EU074639	EVG 06	USA, Florida
*Scolomysmelanops*	AF527419	KU 158213	Perú, San Jacinto
*Scolomysjuruaense*	AF108696	INPA 2489	Brazil, Río Jurua
*Scolomysucayalensis*	EU579518	AMNH 272721	Peru. Loreto
*Thomasomysbaeops*	KR818876	TEL 1960	Ecuador, Carchi
*Thomasomysdaphne*	AF108673	MVZ 171502	Peru, Puno
*Thomasomysischyurus*	AF108675	MVZ 182003	Peru, Cajamarca
*Thomasomyskalinowskii*	AF108678	MVZ 172598	Peru, Junin
*Thomasomysparamorum*	KR818893	TEL 2380	Ecuador, Carchi
*Thomasomystaczanowskii*	KR818885	TEL 2388	Ecuador, Carchi
*Thomasomysvulcani*	KR818904	TEL 2746	Ecuador, Carchi
INGROUP:			
*Neacomyselieceri*	MT462054	JUR 19	Brazil, Pará
*Neacomyselieceri*	MT462055	UFPA 1413	Brazil, Pará
*Neacomyselieceri*	MT462056	JUR 042	Brazil, Pará
*Neacomyselieceri*	MT462057	MPEG 42901	Brazil, Pará
*Neacomysmarajoara*	KX752072	MPEG 40443	Brazil, Pará
*Neacomysmarajoara*	KX752074	MEPG 40439	Brazil, Pará
*Neacomysmarajoara*	KX752075	MPEG 40440	Brazil, Pará
*Neacomysmarajoara*	KX752080	MPEG 40446	Brazil, Pará
*Neacomysmarajoara*	MT462067	MPEG 40441	Brazil, Pará
*Neacomysmarajoara*	MT462068	MPEG 40435	Brazil, Pará
*Neacomysmarajoara*	MT462069	MPEG 40434	Brazil, Pará
*Neacomysmarajoara*	MT462070	MPEG 40432	Brazil, Pará
*Neacomysmarajoara*	MT462071	MPEG 40431	Brazil, Pará
*Neacomysmarajoara*	MT462072	MPEG 40429	Brazil, Pará
*Neacomysvossi*	MT462024	UFPA 1277	Brazil, Pará
*Neacomysvossi*	MT462025	UFPA 1284	Brazil, Pará
*Neacomysvossi*	MT462026	UFPA 1647	Brazil, Pará
*Neacomysvossi*	MT462027	UFPA 1467	Brazil, Pará
*Neacomysvossi*	MT462028	UFPA 1583	Brazil, Pará
*Neacomysvossi*	MT462029	UFPA 1577	Brazil, Pará
*Neacomysvossi*	MT462030	UFPA 1691	Brazil, Pará
*Neacomysvossi*	MT462031	UFPA 1520	Brazil, Pará
*Neacomysvossi*	MT462032	UFPA 1654	Brazil, Pará
*Neacomysvossi*	MT462033	UFPA 1487	Brazil, Pará
*Neacomysvossi*	MT462073	UFPA 1736	Brazil, Pará
*Neacomysvossi*	MT462074	UFPA 1444	Brazil, Pará
*Neacomysvossi*	MT462075	UFPA 1391	Brazil, Pará
*Neacomysxingu*	KX752073	MPEG 41804	Brazil, Pará
*Neacomysxingu*	KX752076	MPEG 41805	Brazil, Pará
*Neacomysxingu*	KX792080	USNM MDC 593	Brazil, Pará
*Neacomysxingu*	MG262333	MZUSP 29540	Brazil, Mato Grosso
*Neacomysxingu*	MT462058	UFMT 1275	Brazil, Pará
*Neacomysxingu*	MT462059	UFMT 1273	Brazil, Pará
*Neacomysxingu*	MT462060	UFMT 1268 i	Brazil, Pará
*Neacomysxingu*	MT462061	MPEG 41805	Brazil, Pará
*Neacomysxingu*	MT462062	MPEG 42019	Brazil, Pará
*Neacomysxingu*	MT462063	MPEG 41996	Brazil, Pará
*Neacomysxingu*	MT462064	PSA 69	Brazil, Pará
*Neacomysxingu*	MT462065	MPEG 41991	Brazil, Pará
*Neacomysxingu*	MT462066	PSA 46	Brazil, Pará
*Neacomysparacou*	FM210765	V 1097	Guyana, Saul
*Neacomysparacou*	FM210766	ROM 114143	Suriname, Brokopongo
*Neacomysparacou*	FM210767	ROM 114315	Suriname, Brokopongo
*Neacomysparacou*	FM210768	ROM 114317	Suriname, Brokopongo
*Neacomysparacou*	FM210769	ROM 114324	Suriname, Brokopongo
*Neacomysparacou*	FM210770	V 1689	Guyana, Mont St. Marcel
*Neacomysparacou*	FM210782	ROM 114325	Suriname, Brokopongo
*Neacomysparacou*	FM210783	V 2002	Guyana, Caiman
*Neacomysparacou*	FM210784	V 1702	Guyana, Nouragues
*Neacomysparacou*	KP778279	ROM 114317	Suriname, Brokopongo
*Neacomysparacou*	KP778309	ROM 114143	Suriname, Brokopongo
*Neacomysparacou*	KP778398	ROM:114150	Suriname, Brokopongo
*Neacomysparacou*	KP778425	ROM 114023	Suriname, Brokopongo
*Neacomysparacou*	KX752077	MPEG 39998	Brazil, Pará
*Neacomysparacou*	KX752078	IEPA 2466	Brazil, Amapa
*Neacomysparacou*	KX792078	ROM 101026	Guyana, Barima-Waini
*Neacomysparacou*	KX792079	ROM 101114	Guyana, Barima-Waini
*Neacomysparacou*	MT462042	CN 279	Brazil, Pará
*Neacomysparacou*	MT462043	CN 263	Brazil, Pará
*Neacomysparacou*	MT462044	CN 186	Brazil, Pará
*Neacomysparacou*	MT462045	CN 184	Brazil, Pará
*Neacomysparacou*	MT462046	CN 129	Brazil, Pará
*Neacomysparacou*	MT462047	CN 70	Brazil, Pará
*Neacomysparacou*	MT462048	CN 66	Brazil, Pará
*Neacomysparacou*	MT462049	INPA 7089	Brazil, Amazonas
*Neacomysparacou*	MT462050	INPA 7138	Brazil, Amazonas
*Neacomysauriventer*	MW512656	MEPN 11863	Ecuador, Zamora Chinchipe
*Neacomysauriventer*	MW512657	MEPN 11870	Ecuador, Zamora Chinchipe
*Neacomysauriventer*	MW512658	MEPN 12079	Ecuador, Zamora Chinchipe
*Neacomysauriventer*	MW512659	MPEN 12086	Ecuador, Zamora Chinchipe
*Neacomysauriventer*	MW512660	MPEN 12110	Ecuador, Zamora Chinchipe
*Neacomysauriventer*	MW512661	MEPN 12306	Ecuador, Zamora Chinchipe
*Neacomysauriventer*	MW512662	MEPN 12312	Ecuador, Zamora Chinchipe
*Neacomysserranensis**	MT536172	UIS-MHN-M 1608	Colombia, Santander
*Neacomysserranensis*	MT536173	UIS-MHN-M 1928	Colombia, Santander
*Neacomysamoenus*	AF108701	MVZ 155015	Peru, Amazonas
*Neacomysamoenus*	GU126521	MVZ 155014	Peru, Amazonas
*Neacomysamoenus*	JQ966232	UFES 1730	Brazil, Mato Grosso
*Neacomysamoenus*	KX792021	LHE 1417	Bolivia, Santa Cruz
*Neacomysamoenus*	KX792022	USNM 584543	Bolivia, Santa Cruz
*Neacomysamoenus*	KX792023	LHE 1558a	Bolivia, Santa Cruz
*Neacomysamoenus*	KX792024	MZ-USP/CIT 371	Brazil, Mato Grosso
*Neacomysamoenus*	KX792025	MZ-USP/CIT 519	Brazil, Mato Grosso
*Neacomysamoenus*	KX792026	MZ-USP/CIT 520	Brazil, Mato Grosso
*Neacomysamoenus*	KX792027	MZ-USP/CIT 534	Brazil, Mato Grosso
*Neacomysamoenus*	KX792028	MZ-USP/CIT 555	Brazil, Mato Grosso
*Neacomysamoenus*	KX792029	MZ-USP/CIT 569	Brazil, Mato Grosso
*Neacomysamoenus*	KX792030	MZ-USP/CIT 665	Brazil, Mato Grosso
*Neacomysamoenus*	KX792031	MZ-USP/CIT 678	Brazil, Mato Grosso
*Neacomysamoenus*	KX792032	INPA 3060	Brazil, Acre
*Neacomysamoenus*	KX792033	INPA 3062	Brazil, Acre
*Neacomysamoenus*	KX792034	MNFS 1263	Brazil, Acre
*Neacomysamoenus*	KX792035	MVZ 193758	Brazil, Acre
*Neacomysamoenus*	KX792036	MVZ 193767	Brazil, Acre
*Neacomysamoenus*	KX792037	MVZ 190364	Brazil, Amazonas
*Neacomysamoenus*	KX792038	MVZ 190365	Brazil, Amazonas
*Neacomysamoenus*	KX792039	MVZ 190367	Brazil, Amazonas
*Neacomysamoenus*	KX792040	MVZ 190372	Brazil, Amazonas
*Neacomysamoenus*	KX792041	USNM 588051	Peru, Cusco
*Neacomysamoenus*	KX792042	USNM 588096	Peru, Cusco
*Neacomysamoenus*	KX792043	MRR 778	Peru, Cusco
*Neacomysamoenus*	KX792044	MRR 799	Peru, Cusco
*Neacomysamoenus*	KX792049	MVZ 155015	Peru, Amazonas
*Neacomysamoenus*	KY859733	MUSM 45055	Peru, Huánuco
*Neacomysamoenus*	KY886320	MUSM 40760	Peru, Junin
*Neacomysamoenus*	KY886321	MUSM 41445	Peru, Junin
*Neacomysamoenus*	KY886322	MUSM 35698	Peru, Loreto
*Neacomysamoenus*	KY886323	MUSM 17990	Peru, Loreto
*Neacomysamoenus*	MG262329	PEU 960057	Brazil, Mato Grosso
*Neacomysamoenus*	MG262330	PEU 960064	Brazil, Mato Grosso
*Neacomysamoenus*	MG262331	M 97024	Brazil, Mato Grosso
*Neacomysamoenus*	MG262332	M 968559	Brazil, Mato Grosso
*Neacomysamoenus*	MT462015	INPA 3059	Brazil, Acre
*Neacomysamoenus*	MT462016	INPA 3064	Brazil, Acre
*Neacomysamoenus*	MT462017	INPA 3063	Brazil, Acre
*Neacomysamoenus*	MT462019	MVZ 190634	Brazil, Amazonas
*Neacomysamoenus*	MT462020	MVZ 190635	Brazil, Amazonas
*Neacomysamoenus*	MT462021	INPA 3057	Brazil, Amazonas
*Neacomysamoenus*	MT462022	USNM 584544	Bolivia, Santa Cruz
*Neacomysamoenus*	MT462076	UFMT 1763	Brazil, Mato Grosso
*Neacomysamoenus*	MT462077	UFMT 1757	Brazil, Mato Grosso
*Neacomysamoenus*	MT462078	UFMT 1669	Brazil, Mato Grosso
*Neacomysamoenus*	MT462079	UFMT 1659	Brazil, Mato Grosso
*Neacomysamoenus*	MT462080	UFMT 1637	Brazil, Mato Grosso
*Neacomysamoenus*	MT462081	UFMT 1374	Brazil, Mato Grosso
*Neacomysamoenus*	MT462082	UFMT 1373	Brazil, Mato Grosso
*Neacomysamoenus*	MT462083	UFMT 1370	Brazil, Mato Grosso
*Neacomysamoenus*	MT462084	INPA-MSANB 46	Brazil, Rondonia
*Neacomysamoenus*	MT462085	INPA-MSAFM 02	Brazil, Rondonia
*Neacomysamoenus*	MT462086	UFMT 3382	Brazil, Mato Grosso
*Neacomysamoenus*	MT462087	UFMT 3380	Brazil, Mato Grosso
*Neacomyscarceleni*	EU579504	MVZ 155014	Peru, Amazonas
*Neacomyscarceleni*	KX792045	ROM 104474	Ecuador, Pastaza
*Neacomyscarceleni*	KX792046	ROM 105278	Ecuador, Pastaza
*Neacomyscarceleni*	KX792047	ROM 105290	Ecuador, Pastaza
*Neacomyscarceleni*	KX792048	USNM 574567	Ecuador, Pastaza
*Neacomyscarceleni*	KY859736	MUSM 45714	Peru, Loreto
*Neacomyscarceleni*	KY859737	PSV 204	Peru, Loreto
*Neacomyscarceleni*	KY859738	ROM 105282	Ecuador, Pastaza
*Neacomyscarceleni*	MT462018	ROM 105264	Ecuador, Napo
*Neacomyscarceleni*	MW512665	QCAZ 10364	Ecuador, Orellana
*Neacomyscarceleni*	MW512666	QCAZ 15251	Ecuador, Orellana
*Neacomyscarceleni*	MW512667	QCAZ 15253	Ecuador, Orellana
*Neacomyscarceleni*	MW512668	QCAZ 15254	Ecuador, Orellana
*Neacomyscarceleni*	MW512669	QCAZ 15805	Ecuador, Orellana
*Neacomyscarceleni*	MW512670	QCAZ 15807	Ecuador, Orellana
*Neacomyscarceleni*	MW512671	QCAZ 15813	Ecuador, Orellana
*Neacomyscarceleni*	MW512672	QCAZ 15814	Ecuador, Orellana
*Neacomyscarceleni*	MW512673	QCAZ 15815	Ecuador, Orellana
*Neacomyscarceleni*	MW512674	QCAZ 15817	Ecuador, Orellana
*Neacomyscarceleni*	MW512675	QCAZ 16124	Ecuador, Orellana
*Neacomyscarceleni*	MW512676	QCAZ 16125	Ecuador, Orellana
*Neacomyscarceleni*	MW512677	QCAZ 16404	Ecuador, Orellana
*Neacomyscarceleni*	MW512678	QCAZ 16406	Ecuador, Orellana
*Neacomyscarceleni*	MW512679	QCAZ 16407	Ecuador, Orellana
*Neacomyscarceleni*	MW512680	QCAZ 16408	Ecuador, Orellana
*Neacomyscarceleni*	MW512681	QCAZ 16412	Ecuador, Orellana
*Neacomyscarceleni*	MW512682	QCAZ 16422	Ecuador, Orellana
*Neacomyscarceleni*	MW512683	QCAZ 16423	Ecuador, Orellana
*Neacomyscarceleni*	MW512684	QCAZ 16424	Ecuador, Orellana
*Neacomyscarceleni*	MW512685	QCAZ 4614	Ecuador, Tungurahua
*Neacomyscarceleni*	MW512686	QCAZ 5308	Ecuador, Pastaza
*Neacomyscarceleni*	MW512687	QCAZ 7013	Ecuador, Sucumbíos
*Neacomyscarceleni*	MW512688	QCAZ 7182	Ecuador, Sucumbíos
*Neacomyscarceleni*	MW512689	QCAZ 7249	Ecuador, Sucumbíos
*Neacomyscarceleni*	MW512690	QCAZ 7251	Ecuador, Sucumbíos
*Neacomyscarceleni*	MW512691	QCAZ 8163	Ecuador, Orellana
*Neacomyscarceleni*	MW512692	QCAZ 8827	Ecuador, Morona Santiago
*Neacomyscarceleni*	MW512693	QCAZ 8828	Ecuador, Morona Santiago
*Neacomyscarceleni*	MW512694	QCAZ 8832	Ecuador, Morona Santiago
*Neacomyscarceleni*	MW512695	QCAZ 8859	Ecuador, Morona Santiago
*Neacomyscarceleni*	MW512696	QCAZ 15818	Ecuador, Orellana
*Neacomysspinosus*	KX258228	MUSM 36924	Peru, Amazonas
*Neacomysspinosus*	KY886327	MUSM 36928	Peru, Amazonas
*Neacomysvargasllosai*	KX258225	MUSM 35076	Peru, Puno
*Neacomysvargasllosai*	KX258226	MUSM 35080	Peru, Puno
*Neacomysvargasllosai*	KX258227	MUSM 35083	Peru, Puno
*Neacomysvargasllosai*	KX792082	MVZ 172650	Peru, Puno
*Neacomysvargasllosai*	MT462013	MVZ 172654	Peru, Puno
*Neacomysvargasllosai*	MT462014	MVZ 172655	Peru, Puno
*Neacomysaletheia*	KX792064	INPA 3050	Brazil, Amazonas
*Neacomysaletheia**	KX792066	INPA 3891	Brazil, Amazonas
*Neacomysaletheia*	KX792067	MPEG/JUR 3	Brazil, Amazonas
*Neacomysaletheia*	KX792070	MVZ 190362	Brazil, Amazonas
*Neacomysaletheia*	KY754054	MVZ 193750	Brazil, Amazonas
*Neacomysaletheia*	KY886324	MUSM 15994	Peru, Loreto
*Neacomysaletheia*	KY886325	MUSM 15993	Peru, Loreto
*Neacomysaletheia*	KY886326	INPA 3056	Brazil, Amazonas
*Neacomysaletheia*	MT462011	INPA 3055	Brazil, Amazonas
*Neacomysaletheia*	MT462012	INPA 3053	Brazil, Amazonas
*Neacomysguianae*	FM210778	CM 76847	Suriname, Nickerie
*Neacomysguianae*	FM210779	CM 76849	Suriname, Saramacca
*Neacomysguianae*	MT462037	INPA 7102	Brazil, Amazonas
*Neacomysguianae*	MT462038	INPA 7100	Brazil, Amazonas
*Neacomysjau*	KX792059	MNFS 2017	Brazil, Amazonas
*Neacomysjau*	KX792060	MNFS 2023	Brazil, Amazonas
*Neacomysjau*	KX792061	MNFS 2084	Brazil, Amazonas
*Neacomysjau*	KX792062	MNFS 2104	Brazil, Amazonas
*Neacomysjau*	MT462051	MPEG 45483	Brazil, Amazonas
*Neacomysmacedoruizi*	KY859731	MUSM 45054	Peru, Huánuco
*Neacomysmacedoruizi**	KY859732	MUSM 45053	Peru, Huánuco
*Neacomysmarci* sp. nov.	Pending	QCAZ 18677	Ecuador, Pichincha
*Neacomysmarci* sp. nov.	Pending	MECN 5339	Ecuador, Carchi
*Neacomysmarci* sp. nov.	Pending	MECN 5340	Ecuador, Carchi
*Neacomysmarci* sp. nov.	Pending	MECN 7569	Ecuador, Esmeraldas
*Neacomysmarci* sp. nov.	Pending	MECN 7564	Ecuador, Esmeraldas
*Neacomysmarci* sp. nov.	Pending	MECN 7568	Ecuador, Esmeraldas
*Neacomystenuipes**	KX792081	BM 1899.10.3.34	Colombia, Cundinamarca
*Neacomystenuipes*	MT536165	UIS-MHN-M 927	Colombia, Santander
*Neacomystenuipes*	MT536169	MHN-Uca 1627	Colombia, Caldas
*Neacomystenuipes*	MT536170	MHN-Uca 1628	Colombia, Caldas
*Neacomystenuipes*	MT543038	MPIII 32	Colombia, Antioquia
*Neacomystenuipes*	MT536171	MHN-UCa 1761	Colombia, Caldas
*Neacomystenuipes*	MT536166	UIS-MHN-M 1720	Colombia, Bolívar
*Neacomystenuipes*	MT536167	UIS-MHN-M 1723	Colombia, Bolívar
*Neacomystenuipes*	MT536168	UIS-MHN-M 1762	Colombia, Bolívar
*Neacomystenuipes*	MT543037	CTUA 2599	Colombia, Antioquia
*Neacomysminutus* s. s.	KX792063	INPA 3047	Brazil, Amazonas
*Neacomysminutus* s. s.	KX792065	INPA 3051	Brazil, Amazonas
*Neacomysminutus* s. s.	KX792068	MVZ 190359	Brazil, Amazonas
*Neacomysminutus* s. s.	KX792069	MVZ 190361	Brazil, Amazonas
*Neacomysminutus* s. s.	KX792071	MVZ 190363	Brazil, Amazonas
*Neacomysminutus* s. s.	KX792072	MVZ 191209	Brazil, Amazonas
*Neacomysminutus* s. s.	KY859739	MVZ 190360	Brazil, Amazonas
*Neacomysminutus* s. s.	KY859740	MNFS 1734	Brazil, Amazonas
*Neacomysminutus* s. s.	KY859741	MNFS 1787	Brazil, Amazonas
*Neacomysminutus* s. s.	MT462008	INPA 3048	Brazil, Amazonas
*Neacomysminutus* s. s.	MT462009	INPA 3049	Brazil, Amazonas
*Neacomysminutus* s. s.	MT462010	INPA 2689	Brazil, Amazonas
*Neacomysmusseri*	EU579503	AMNH 272676	Peru, Loreto
*Neacomysmusseri*	KX792074	MVZ 171487	Peru, Cusco
*Neacomysmusseri*	KX792076	AMNH 272687	Peru, Loreto
*Neacomysmusseri*	KX792077	KU 144300	Peru, Madre de Dios
*Neacomysmusseri*	KY754055	MVZ 193763	Brazil, Acre
*Neacomysmusseri*	KY859742	MVZ 171488	Peru, Cusco
*Neacomysrosalindae*	KX792050	ROM 104560	Ecuador, Napo
*Neacomysrosalindae*	KX792051	ROM 105265	Ecuador, Napo
*Neacomysrosalindae*	KX792052	ROM 105314	Ecuador, Napo
*Neacomysrosalindae*	KX792053	ROM 105315	Ecuador, Napo
*Neacomysrosalindae*	KX792054	MVZ 153530	Perú, Amazonas
*Neacomysrosalindae*	KX792055	KU 158172	Peru, Loreto
*Neacomysrosalindae*	KX792056	TK 73307	Peru, Loreto
*Neacomysrosalindae*	KX792057	TK 73347	Peru, Loreto
*Neacomysrosalindae*	KX792058	TK 73493	Peru, Loreto
*Neacomysrosalindae*	KY826416	MUSM 45717	Peru, Loreto
*Neacomysrosalindae*	KY859730	MVZ 155299	Peru, Amazonas
*Neacomysrosalindae*	KY859743	MUSM 45720	Peru, Loreto
*Neacomysrosalindae*	KY859744	MUSM 45719	Peru, Loreto
*Neacomysrosalindae*	KY859745	MUSM 45718	Peru, Loreto
*Neacomysrosalindae*	KY859746	MUSM 45721	Peru, Loreto
*Neacomysrosalindae*	KY859747	MUSM 45731	Peru, Loreto
*Neacomysrosalindae*	KY859748	MUSM 45716	Peru, Loreto
*Neacomysrosalindae*	KY859749	MUSM 45730	Peru, Loreto
*Neacomysrosalindae*	KY859750	MUSM 45733	Peru, Loreto
*Neacomysrosalindae*	KY859751	MUSM 45729	Peru, Loreto
*Neacomysrosalindae*	KY859752	MUSM 45728	Peru, Loreto
*Neacomysrosalindae*	KY859753	MUSM 45727	Peru, Loreto
*Neacomysrosalindae*	KY859754	MUSM 45734	Peru, Loreto
*Neacomysrosalindae*	KY859755	MUSM 44964	Peru, Loreto
*Neacomysrosalindae*	KY859756	MUSM 44967	Peru, Loreto
*Neacomysrosalindae*	KY859757	MUSM 44966	Peru, Loreto
*Neacomysrosalindae*	KY859758	MUSM 44968	Peru, Loreto
*Neacomysrosalindae*	KY859759	MUSM 44972	Peru, Loreto
*Neacomysrosalindae*	KY859760	MUSM 44969	Peru, Loreto
*Neacomysrosalindae*	KY859761	MUSM 44971	Peru, Loreto
*Neacomysrosalindae**	KY859762	MUSM 44963	Peru, Loreto
*Neacomysrosalindae*	KY859763	VPT 4794	Peru, Loreto
